# Cheaper faster drug development validated by the repositioning of drugs against neglected tropical diseases

**DOI:** 10.1098/rsif.2014.1289

**Published:** 2015-03-06

**Authors:** Kevin Williams, Elizabeth Bilsland, Andrew Sparkes, Wayne Aubrey, Michael Young, Larisa N. Soldatova, Kurt De Grave, Jan Ramon, Michaela de Clare, Worachart Sirawaraporn, Stephen G. Oliver, Ross D. King

**Affiliations:** 1 Department of Computer Science, Aberystwyth University, Aberystwyth SY23 3DB, UK; 2 Cambridge Systems Biology Centre and Department of Biochemistry, University of Cambridge, Sanger Building, 80 Tennis Court Road, Cambridge CB2 1GA, UK; 3 Department of Structural and Functional Biology, UNICAMP, 13083-865, Campinas, São Paulo, Brazil; 4 Institute of Biological, Environmental and Rural Sciences, Aberystwyth University, Aberystwyth SY23 3DD, UK; 5 Department of Computer Science, Brunel University, London UB8 3PH, UK; 6 Department of Computer Science, KU Leuven, 3001 Heverlee, Belgium; 7 Department of Biochemistry, Mahidol University, Thailand; 8 Manchester Institute of Biotechnology and School of Computer Science, University of Manchester, Manchester M1 7DN, UK

**Keywords:** drug design, artificial intelligence, quantitative structure activity relationship

## Abstract

There is an urgent need to make drug discovery cheaper and faster. This will enable the development of treatments for diseases currently neglected for economic reasons, such as tropical and orphan diseases, and generally increase the supply of new drugs. Here, we report the Robot Scientist ‘Eve’ designed to make drug discovery more economical. A Robot Scientist is a laboratory automation system that uses artificial intelligence (AI) techniques to discover scientific knowledge through cycles of experimentation. Eve integrates and automates library-screening, hit-confirmation, and lead generation through cycles of quantitative structure activity relationship learning and testing. Using econometric modelling we demonstrate that the use of AI to select compounds economically outperforms standard drug screening. For further efficiency Eve uses a standardized form of assay to compute Boolean functions of compound properties. These assays can be quickly and cheaply engineered using synthetic biology, enabling more targets to be assayed for a given budget. Eve has repositioned several drugs against specific targets in parasites that cause tropical diseases. One validated discovery is that the anti-cancer compound TNP-470 is a potent inhibitor of dihydrofolate reductase from the malaria-causing parasite *Plasmodium vivax*.

## 1. Introduction

New drugs are generally slow (more than 10 years) and expensive (more than $1 Billion) to discover and develop. Consequently tropical diseases, malaria, schistosomiasis, Chagas' disease, etc., which kill millions of people and infect hundreds of millions of others are ‘neglected’ [1,2]; and ‘orphan’ diseases with few sufferers remain untreatable [3]. More generally, the pharmaceutical industry is struggling to cope with spiralling drug discovery and development costs [4].

**Figure 1.**
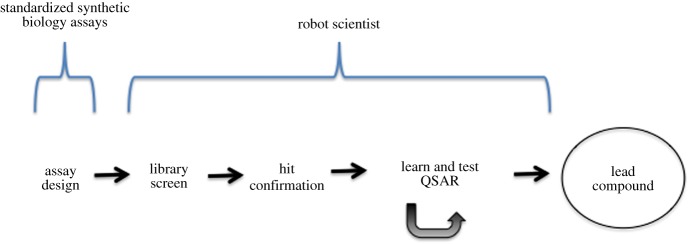
Early stage drug design. The contribution of standardized synthetic biology assays and Eve to a cheaper faster drug discovery pipeline. (Online version in colour.)

The most important steps in early stage drug design are shown in figure 1 [5]. A key initial step is to develop an ‘assay’. This is a ‘wet’ (biological/chemical) or ‘dry’ (computational) experiment that estimates whether a small molecule (compound) is likely to treat a disease. This assay should be relatively cheap and fast to execute as it will be run multiple times. A compound that passes the assay is called a ‘hit’. The next step is to run a drug screen, where a ‘library’ (set) of compounds is tested against the assay. This library may be very large, tens/hundreds of thousands, maybe millions of compounds. Such mass screening is generally done in a brute-force and unintelligent way: ‘begin at the beginning and go on till you come to the end: then stop’ (Lewis Carroll). As the *a priori* probability of any library compound being a hit is low, it is difficult to design an assay that does not have an appreciable number of false positive hits. Therefore, it is generally necessary to execute experiments to retest (‘confirm’) the hits. These experiments are more expensive and slow to execute, but have a much lower false positive probability. From the set of confirmed hit activities, a quantitative structure activity relationship (QSAR) is learnt [6]. This is a function whose input is the structure of a compound, and whose output is the predicted activity on the assay. As the output is typically a real number, QSAR learning is generally a regression task. QSARs generalize the results of assays and guide the synthesis of new compounds. After new compounds are synthesized, they are tested against the hit-confirmation assay, and the results of these assays are used to learn a more accurate QSAR, and the cycle repeated. The process is terminated when a compound is found that has a sufficiently high score on the assay, and which passes other tests such as low predicted toxicity, potential for modification, etc. This compound is called a ‘lead’.

The standard way to improve the economics of a process is through automation and standardization [7]. The use of automation has been enthusiastically pursued by the pharmaceutical industry. Much of this effort has gone into making library screening faster, especially through miniaturization, with the result that high-throughput robotic systems now routinely screen millions of compounds in library screens [5]. Less effort has gone into automating other steps of early stage drug design, and standardization has been little used.

A natural extension of the trend of increased involvement of automation in science is the concept of a Robot Scientist [8,9]. A Robot Scientist automatically: originates hypotheses to explain observations, devises experiments to test these hypotheses, physically runs the experiments using laboratory robotics, interprets the results to change the probability of hypotheses, and then repeats the cycle. In this way Robot Scientists can automate high-throughput hypothesis led research. Robot Scientists are also well suited to recording scientific knowledge: as the experiments are conceived and executed automatically by computer, it is possible to completely capture and digitally curate all aspects of the scientific process. [9]. The first Robot Scientist ‘Adam’ was designed to plan and execute yeast microbiological experiments. Adam was fully automated and during an investigation there was no essential requirement for a technician, except to periodically add laboratory consumables and remove waste. Adam was the first machine demonstrated to have autonomously discovered novel scientific knowledge [9]. Adam investigated the functional genomics of *S. cerevisiae* and discovered the function of locally orphan enzymes—enzymes known to be in yeast but for which the gene(s) encoding them are unknown [9]. The advances that distinguished Adam from other complex laboratory systems (such as high-throughput drug-screening pipelines and X-ray crystallography crystal-screening systems) was its artificial intelligence (AI) software, its many complex internal cycles, and its ability to execute high-throughput individually planned cycles of experiments.

In this paper, we demonstrate the viability of the Robot Scientist ‘Eve’ in drug discovery. We focus on finding lead compounds for neglected tropical diseases. However, the principles and methods used can be generally employed. The reasons for the focus on neglected tropical diseases are:

— These diseases are a scourge of humanity, infecting hundreds of millions of people, and annually killing millions of people.
— The aetiology of these diseases is clear, as is what needs to be done to treat them (kill the parasites), and how to achieve this treatment with a small molecule drug. These criteria are not met for many diseases targeted by the Pharmaceutical Industry.
— There is little competition from the much better funded Pharmaceutical Industry.

## 2. Eve

### 2.1. Design

**Figure 2.**
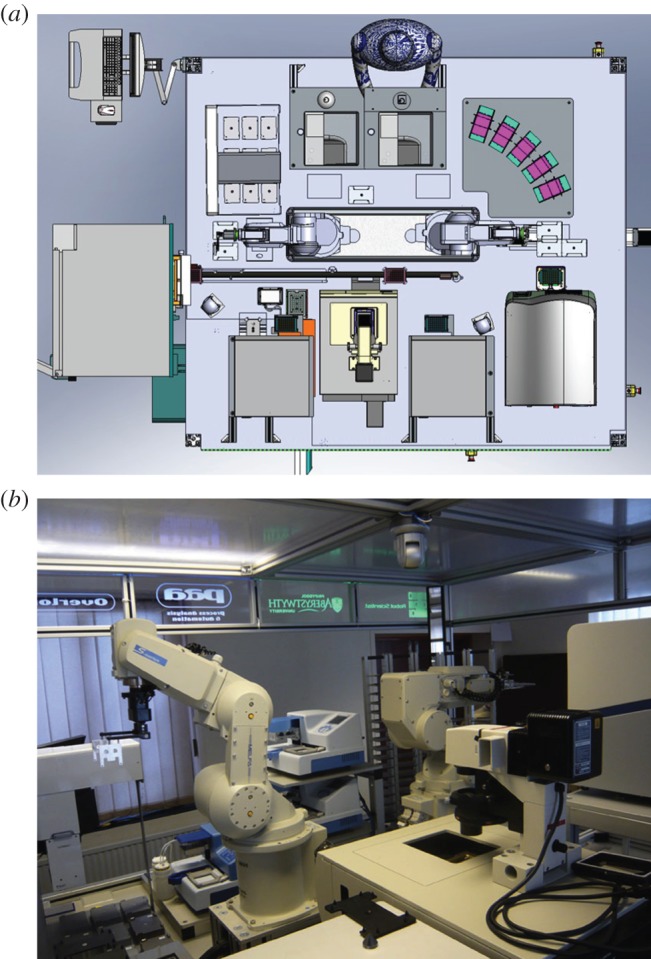
(*a*) A diagram of Eve. Showing the location of the main instruments. (*b*) A photo of Eve. Eve has been designed to be flexible in terms of the biological assays that it can perform, and is physically capable of screening at a moderately high-throughput rate.

We report the development of the Robot Scientist Eve designed to automate early stage drug design (figure 2). The initial design of Eve was given in [10]. Eve has three integrated modes corresponding to successive stages in lead drug discovery. In its Library-screening mode, Eve systematically tests each member from a large set of compounds against an assay in the standard brute-force way of conventional mass screening [5]. While simple to automate, brute-force mass screening is slow and wasteful of resources as every compound in the library is tested. It is also unintelligent, as it makes no use of what is learnt during screening. Eve starts the lead discovery process by mass-screening a subset of its library to find ‘hit’ compounds for the assay. This subset is currently chosen randomly.

In its hit-confirmation mode Eve re-assays the hit compounds using multiple repeats and titrations to reduce the probability of false positives. Eve's integration of screening and hit-confirmation is similar to advanced screening systems that first execute a high-throughput screen, and then a high-content screen for selected compounds.

Starting from the set of confirmed hits, Eve executes cycles of statistics/machine learning that hypothesize QSARs, and tests these QSARs on new compounds. As Eve currently does not have access to chemical synthesis automation [11], we applied Eve to screen untested compounds from its library in lieu of synthesizing compounds. Such intelligent library screening may be more economical than standard mass screening as it potentially saves on time and compound use.

### 2.2. Hardware

Eve's robotic system is capable of moderately high-throughput compound screening: greater than 10 000 compounds per day, depending on the length of time taken to assay compounds. Eve is designed to be sufficiently flexible that it can be rapidly reconfigured to carry out a number of different biological assays, using fluorescence, absorbance or cell morphology as read-outs (figure 2). Eve's robotic system integrates a range of off-the-shelf pieces of laboratory automation equipment into a single system that can perform library-screening, hit-confirmation and cycles of QSAR hypothesis formation and testing using selected compounds from a compound library. Eve can also be reconfigured to copy compound libraries.

Eve's compound library is maintained in a dry-store, with the compounds dissolved in DMSO. Compounds to be assayed are transferred from the storage plates to the assay plates using a non-contact acoustic transfer liquid-handling system. This has the advantages of high accuracy and saving pipette tips. In library-screening mode there is a direct mapping from storage plates to assay plates, and a single transfer volume is used in each well. In hit-confirmation and intelligent-screening modes a single compound from the storage plate is transferred to multiple wells in the assay plate, and at different volumes, to realize multiple repeats dose–response experiments. After the addition of assay compound, the target yeast strain pool is added using a simple liquid-handling robot, as the same volume is added to each well. The yeast pool is created externally and stored by Eve for use. Once the assay plates are formed they are placed in a shaking incubator. Every 90 min, the plates are removed from the incubator, and fluorescence measured. Eve has two microplate readers capable of recording measurements across a broad range of both excitation and emission wavelengths. Eve also has an automated microscope capable of taking both bright-field and fluorescence images across a broad range of wavelengths. Upon completion of the assay, the plates are automatically removed from the system. To transfer the plates between different pieces of laboratory automation equipment, Eve uses robotic arms and linear actuators. All plates are bar-coded and movements recorded.

### 2.3. Low-level software

Software was written to integrate Eve's AI software with the robotics and thereby automate and integrate Eve's early stage drug design functions: library-screening, hit-confirmation and QSAR cycles. The software to control the robotics, instrumentation and used to execute the experiments was written on top of Peak Analysis and Automation's Overlord software. An interface was written to a relational database that stores all experiment-related data and meta-data, e.g. all fluorescence measurements. The software to parametrize growth curves for the different yeast strains in each well was taken from the Adam project [9]. The main parameters are estimated maximum growth, doubling time and lag time. These growth parameters were then transferred to the AI QSAR software. Software was also written to coordinate library-screening, and to plan hit-confirmation. This was also integrated with the AI software so that the active learning algorithm could select compounds to test.

### 2.4. Automated quantitative structure activity relationship formation

To form QSARs Eve uses least-squares linear regression with mild 2-norm regularization (ridge regression). This can be interpreted as a Gaussian process with a linear kernel [12], hence we can compute the posterior uncertainty, allowing us to use an optimization method which is more efficient, i.e. which needs fewer function evaluations [13]. The linear kernel choice has the distinct advantage that it permits more efficient computation than other kernels when the dimensionality of the feature space is smaller than the number of examples. The feature space consists of binary fingerprints of all paths up to length 7. We computed these with Open Babel [14].

### 2.5. Active learning

To select compounds to test its hypotheses Eve uses active learning [13,15]. The active learning task is comparable to that in many other areas of science and engineering: identify or design artefacts that have optimal performance. However, it has an extra ingredient reminiscent of reinforcement learning: balancing the *exploration* of compound space with the *exploitation* of regions of highly active compounds. Another complication is that it is desirable to identify the *K* best diverse compounds in the library: ‘leads’ [13]. Therefore, the QSAR active learning problem is: given a finite pool *P* of instances, an unknown QSAR function *f* that maps instances *x* ∈ *P* to their target values *f*(*x*), an assay (noisy ‘oracle’) that can be queried for the target value of any *x* in batches of *N*, the number *K* of leads required; then find the top *K* leads in *P*. (In computer science, an ‘oracle’ is a machine, the workings of which are unexplained, which always returns the correct answer to a question. A noisy oracle has a probability of returning an incorrect answer.) We found a successful approach to be a combination of selecting compounds with high estimated activity T, and high estimated variance, i.e. select the example where 
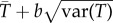
 is maximal [13] (electronic supplementary material). As it is generally inefficient to assay (or synthesize), a single compound in a QSAR cycle, batches of N compounds should be selected (for Eve, *N* = 64). Like the requirement to find the K best leads this greatly increases the computational complexity of choosing the best experiment. Therefore, Eve adopts a greedy strategy to select batch compounds.

## 3. Standardized assays

There are three main forms of assay: computational [16], biochemical and cell-based [17]. The most general type is computational (*in silico* screening, [18])—assuming the Church–Turing thesis [19], they could compute any conceivable assay. The advantages of *in silico* screening are that it is cheap, fast, and that compounds can be tested without synthesizing them. These enable very large libraries to be evaluated, and hence *in silico* screening has proved its worth many time [18]. The main disadvantage of *in silico* screening is that it is computationally infeasible to simulate the full complexity of biological systems. Biochemical assays have the advantage of being target-based (enabling rational drug design), but often assume a specific mechanism of interaction, and provide little information about toxicity, drug uptake into cells and *in vivo* activity. Cell-based assays are the most biologically realistic, but are rarely target-based, and thus provide limited information on the mechanism of action of a drug. Moreover, cell-based assays are not applicable when searching for compounds active against parasites that are not currently possible or difficult to culture (e.g. *Plasmodium vivax*). All these types of assay are slow and expensive to develop—even computational ones if reasonable realism is to be achieved.

**Figure 3.**
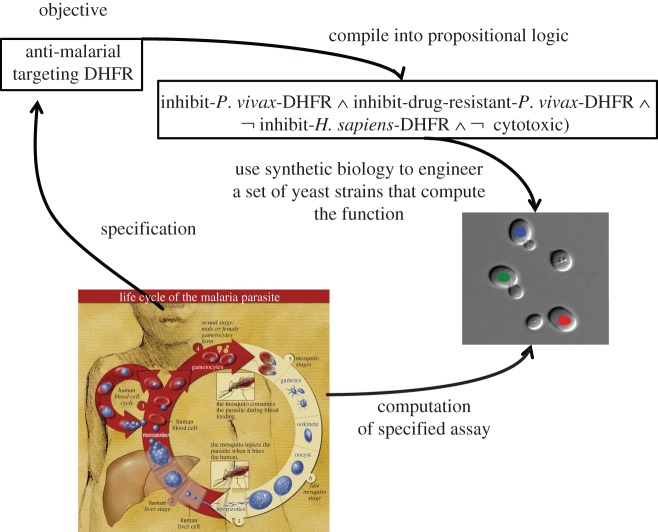
The form of the standardized assays. From biological knowledge, a specific objective is determined; this is compiled into a propositional logic function, and synthetic biology is used to engineer a set of yeast strains that compute the function. (Colour, Medical Illustration, Source: National Institute of Allergy and Infectious Diseases (NIAID), Date Created: 2007, Date Added: 8/20/2013.)

We have developed a standardized form of screening assay that combines advantages of computational assays (generality), biochemical assays (targeted) and using live cells (biological realism, and early screening for toxicity) (figure 3). These assays are designed to be automatically engineered using existing laboratory automation, and can be generated much faster and more cheaply than the bespoke assays that are currently standard. This enables more types of assay to be executed, more efficient use of screening facilities to be made, and thereby increases the probability of a discovery within a given budget. The assays are biological systems designed to compute Boolean functions of desired properties [20]. This concept generalizes previous uses of engineered cells in drug discovery assays [21–23].

As an illustration, consider the example of designing an assay that targets both wild-type and pyrimethamine-resistant (drug-resistant) *P. vivax* dihydrofolate reductases (DHFRs) (figure 3). To compute this function, we first engineer a chimeric yeast (*Saccharomyces cerevisiae*) strain with its DHFR coding sequence (cds) replaced by that for wild-type *P. vivax* DHFR (y^Pv^DHFRp**)**, then engineer a second chimeric yeast strain (y^Pv^Rdhfrp**)** with its DHFR cds replaced by that for drug-resistant *P. vivax* DHFR. We then engineer a third chimeric yeast strain (y^Hs^DHFRp**)** with its DHFR cds replaced by that for *H. sapiens* DHFR. Finally, we apply this biological system to assay for compounds that inhibit growth of the strains expressing the parasite targets (y^Pv^DHFRp and y^Pv^Rdhfrp) and not the strain expressing their human counterpart (y^Hs^DHFRp) [24]. Such compounds are ‘true’ for the assay. They are unlikely to be cytotoxic, as that would imply that the yeast strain expressing the human enzyme would also be inhibited. However, this does not completely remove the probability of human cytotoxicity as there could be off target effects specific to human cells, therefore further studies are required. In practice, Eve grows the strains in competition, in mixed cultures and in 384-well microtitre plates [23] in the presence of one compound from its library. The whole system is a *model* of what we really are interested in: the *in vivo* survival of wild-type/drug-resistant *P. vivax* cells versus those of its human host*.* It can be seen that a set of genetically engineered yeast strains can compute arbitrarily complex Boolean functions of desired assay properties.

## 4. Drug screening and repositioning

### 4.1. Standardized assays

We first demonstrated that we could efficiently generate standardized assays. We generated assays targeting DHFRs (wild-type and pyrimethamine-resistant), *N*-myristoyltransferase (NMT) and phosphoglycerate kinase (PGK) from multiple parasitic organisms: *Trypanosoma brucei* (African sleeping sickness), *Trypanosoma cruzi* (Chagas disease), *Leishmania major* (Leishmaniasis) and *Schistosoma mansoni* (Schistosomiasis) (electronic supplementary material). These assays were much faster and cheaper to develop than using standard methods of assay development: engineering each assay took about one person-month, and cost approximately $15 k. A subset of these assays were reported in [23].

### 4.2. Drug screening

We then tested the utility of these assays, and the efficiency of Eve at standard screening, i.e. running in its library-screening and hit-confirmation modes (table 1). We ran the Maybridge Hitfinder library of approximately 14 400 chemically diverse compounds to these assays. This identified numerous hits. A subset of these results were reported in [23].

**Table 1.** The targets (disease/species/protein/drug-resistant) and libraries screened (May, Maybridge Hitfinder; JH, Johns Hopkins University Clinical Compound Library).

disease	species	enzyme	drug-resistant	libraries
malaria	*P. falciparum*	DHFR	no	May, JH
malaria	*P. falciparum*	DHFR	yes	May, JH
malaria	*P. falciparum*	DHFR	no	May, JH
malaria	*P. vivax*	DHFR	no	May, JH
malaria	*P. vivax*	DHFR	yes	May, JH
malaria	*P. vivax*	DHFR	no	May, JH
malaria	*P. vivax*	PGK	no	May, JH
malaria	*P. vivax*	NMT	no	May, JH
Chagas	*T. cruzi*	DHFR	no	May, JH
Chagas	*T. cruzi*	PGK	no	May, JH
Chagas	*T. cruzi*	NMT	no	May, JH
African sleeping sickness	*T. brucei*	DHFR	no	May, JH
African sleeping sickness	*T. brucei*	PGK	no	May, JH
African sleeping sickness	*T. brucei*	NMT	no	May, JH
schistosomiasis	*S. mansoni*	DHFR	no	May, JH
schistosomiasis	*S. mansoni*	PGK	no	May, JH
schistosomiasis	*S. mansoni*	NMT	no	May, JH
leishmaniasis	*L. major*	DHFR	no	May, JH
bacterial infection	*S. aureus*	DHFR	no	May, JH

### 4.3. Drug screening for drug repositioning

We then applied the assays to the challenge of drug repositioning—the application of known drugs to new diseases (table 1). To do this, we again used Eve in its library-screening and hit-confirmation modes to screen and confirm hits for the above assays, but using the Johns Hopkins University Clinical Compound Library that contains approximately 1600 FDA-and foreign-approved drugs. Several repositioned compounds were found that discriminate between host and parasite, and have passed initial cytotoxicity tests. To maximize the utility and reuse of these screening data, they are available as open data in Resource Description Framework (RDF) format [25] (electronic supplementary material).

### 4.4. Repositioning TNP-470 as an anti-malaria compound

**Figure 4.**
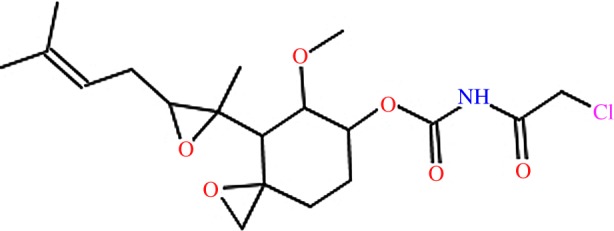
The structure of TNP-470. (Online version in colour.)

**Figure 5.**
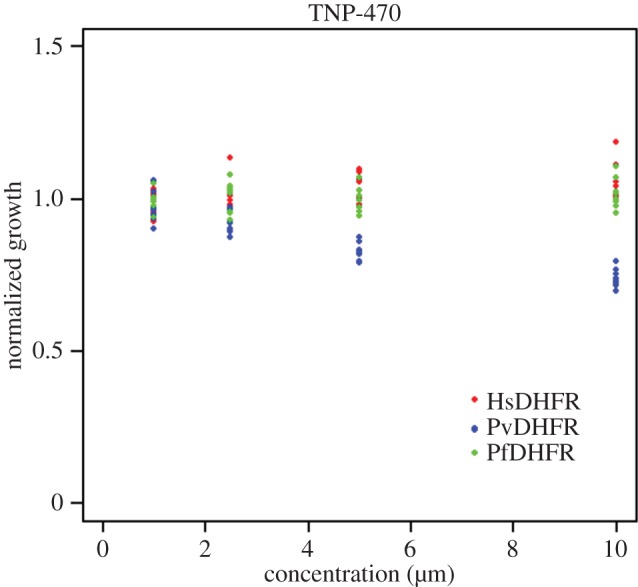
An Eve hit-confirmation run with four replicates. TNP-470 dose response curves for: y^Hs^DHFRp (red), y^Pf^DHFRp (green) and y^Pv^DHFRp (blue). Normalized growth is calculated by comparison to in-plate negative controls.

The compound TNP-470 was derived from the antimicrobial compound fumagillin (figure 4). TNP-470 is an angiogenesis inhibitor (mediated by its irreversible binding to methionine aminopeptidase-2 (MetAP2)) that has been investigated as an anti-cancer drug. TNP-470 and its analogues have been shown to bind to *P. falciparum* MetAP2 *in vitro*, to inhibit growth of *P. falciparum* strains (including the chloroquine-resistant strains W2 and C2B)*,* and to inhibit parasitaemia in a mouse model [26–28]. Eve's yeast synthetic biology assay results indicate that TNP-470 has high activity against *P. vivax* DHFR (figure 5). To further confirm that DHFR is an additional target of TNP-470 we performed DHFR enzyme inhibition assays [29]. We observed that *P. vivax* DHFR was 1000-fold more sensitive to TNP-470 than its human counterpart; the drug's IC_50_ for the parasite enzyme being 0.16 µM, compared to more than 165 µM for human DHFR. This is consistent with the results of Eve's assays and suggests that our approach identified a *bona fide* DHFR inhibitor with improved selectivity.

DHFR inhibitors are currently routinely used as prophylactics against malaria and are given to over a million children in seasonal malaria chemoprevention. However, DHFR inhibitors are no longer used as a standard treatment because of the evolution of drug resistance [6]. Extensive efforts to discover a second-generation DHFR-targeted anti-malarial drug with efficacy against pyrimethamine-resistant strains have yet to produce a compound that has passed clinical trials [30]. Therefore, the discovery of an approved compound with activity against DHFR is of high potential value. It is also significant that TNP-470 is an example of ‘polypharmacology’ [31], in that it targets both *Plasmodium* DHFR and MetAP2. This means that it should be pre-hardened to the evolution of drug resistance, as this would require simultaneous alteration of both targets.

## 5. Automating drug development

### 5.1. Automating drug development

We integrated all three of Eve's modes (library-screening, hit-confirmation, intelligent screening) together to demonstrate that early stage drug development can be automated, including QSAR generate-and-test cycles. The division of labour between Eve and the human scientists and technicians was as follows: the problem task was first tightly defined by the humans who engineered the assays, and defined the QSAR problem. This was the extent of human intellectual effort. Human manual effort was required to maintain and run Eve, maintain consumables, yeast stocks, etc. Human manual effort was also required to run certain programs during the different stages of the cycles, as some of the steps are not fully integrated; these program steps are predetermined, and could if necessary be fully automated.

The first full experimental tests of the active learning loop were conducted by splitting the screened data comprising the heterologous DHFR yeast strains for *P. falciparum, P. vivax*, and that of humans, using 4800 compounds as a training set. The ratio of the yields of the HsDHFR and PvDHFR and PfDHFR strains were passed to the selection algorithm, together with fingerprints of the remaining 9600 compounds. The results from the first ‘cherry-picking’ round (compounds selected by active learning and using the hit-confirmation assays) (*n* = 96; 12 plates of eight compounds per plate; eight replicates of six concentrations) were then added to the original dataset, and a second cherry-picking round conducted. We used these data to evaluate different approaches to the problem of combining cherry-picking and mass-screening data. The approach based on using the mean of replicates multiplied by log(10/conc.) was found to perform best. We then ran the active learning loop through three iterations: an initial set of 4800 compounds was screened (single iteration, 10 µM), and three loops of 96 cherry-picked compounds (eight replicates, at a range of concentrations) were selected. The mean log-weighted cherry-picking data was cycled back into the training set.

### 5.2. Econometric modelling

A thorough investigation of Eve's QSAR active learning methods, comparing intelligent screening versus standard brute-force screening, requires the analysis of thousands of cycles. We therefore decided to use our empirical results from using Eve (in Library-screening mode) against the complete set of 14 400 compounds of the Maybridge HitFinder library against DHFR assays from multiple parasitic organisms (see above)—we considered the Johns Hopkin's library to be too small for intelligent screening. The idea was to use these results as an oracle—instead of new physical experiments. One refinement that we did not investigate was the role of ordering of compounds in the library: we used a constant random order. It would have been interesting to investigate the use molecular of diversity measures to order compounds for screening, this would be expected to find hits faster than random screening. In cases where the target has a known structure, it would have been interesting to investigate *in silico* screening to order compounds as likely hits.

**Figure 6.**
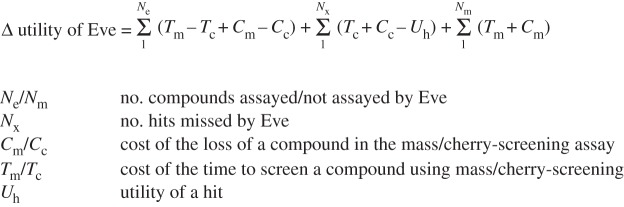
Modelling the economics of drug discovery. The econometric model of the differential utility of intelligent screening versus mass screening with hit-confirmation.

**Figure 7.**
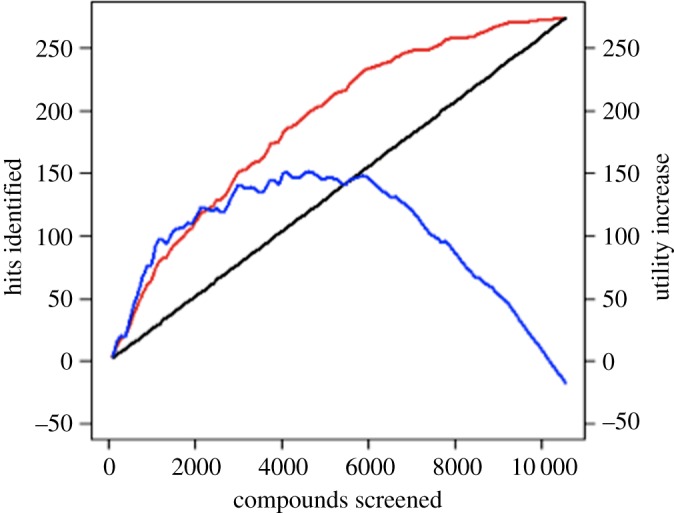
Intelligent versus Random Screening. An example simulation run of intelligent screening: cycles of QSAR learning/testing from a compound library. The data are taken for a screen of the Maybridge Hitfinder library against the *P. vivax* DHFR as target (electronic supplementary material). Intelligent screening is red and standard brute-force black. The differential utility of intelligent screening is shown in blue. It can be seen that it is cost-optimal to screen between a third and a half of Eve's small library, with a larger library the screened proportion would be expected to be smaller. Similar diagrams for the other targets can be found in the electronic supplementary material.

To quantify the utility of intelligent screening, we developed an econometric model (figure 6). In this model the net utility is the cost saving due to not screening compounds, minus the cost due to missing any hits, minus the cumulative cost of the number of active learning cycles performed. Active learning was applied to the seed input data, and predictions made to produce simulated learning curves. The progression of these learning curves was then compared to the base case of standard library screening. For each 96-compound loop, the utility equation was applied. Figure 7 shows the result of one such run involving many cycles of learning and demonstrates hit enrichment by intelligent screening.

**Figure 8.**
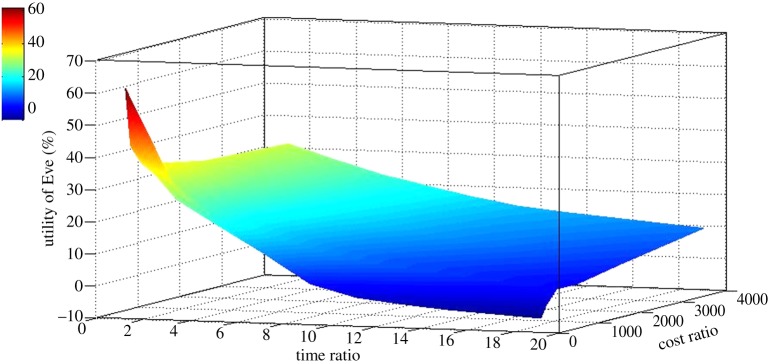
Summary of utility modelling. Diagram of the maximum utility of intelligent screening taken from a systematic scan of different costs/utilities in the econometric model (*a*), using the screening results in (*b*). To make these results comprehensible, we project them down into a three-dimensional graph and combine cost/utilities: time ratio = *T*_c_/*T*_m_ and cost ratio = *U*_h_/*C*_c_. This indicates that intelligent screening is generally rational (there is little area of negative utility), and that a high time-ratio (fast screening) and low cost-ratio (valuable library compounds) are most favourable.

We used the model to investigate a range of costings to determine under what conditions it is economically advantageous compared with performing a standard whole-library screen. Figure 8 shows that under most conditions it is economically rational to screen intelligently. Assuming that the probability of a compound being a hit is independent of the size of the library, i.e. they are independent and identically distributed variables (iid), then the utility gained from intelligent screening is proportional to the size of the library—larger libraries produce larger savings. The iid assumption is reasonable and, in large part, the motivation for the collation of the very large libraries currently used for screening. However, it is also conservative, as the difficulties in physically creating structurally diverse libraries means that the probability of an individual compound being a novel structural hit probably decreases with the size of the library, which means that the savings are probably much greater for large libraries. Therefore, intelligent screening is more cost-effective with larger libraries, more valuable compounds and fast cycles of assay screening and testing—this is the standard regime for pharmaceutical screening, suggesting that adoption of intelligent screening is economically rational.

## 6. Data and code

We developed a semantic data model of Eve's-screening assay results (see electronic supplementary material), where the root node ‘assay triple screen’ represents the main group of data items used to analyse the results. This root node is linked to the node ‘Eve’ via the relation *ro:has-agent*. The semantics of this association are that Eve initiates and runs the process ‘assay triple screen’. The assay triple screen process has the following inputs (*ro: has-input*): synthetic yeast strain(s), each has a unique identifier and *ro:has-part* fluorophore and DHFR target; compound is represented by SMILES code and *sio:has-identifier* compound common name and Maybridge hit finder ID; plate is represented by a code and *ro:has-part* well-column and well-row to identify each well. The semantics of these associations are that synthetic yeast strains, compounds and a plate participate in the assay triple screen process and are present at the beginning of the process. The assay triple screen process has the following outputs (*ro: has-output*): venus, sapphire and cherry initial fluorescence in a well; venus, sapphire and cherry final fluorescence in a well; venus, sapphire and cherry doubling time in a well; venus, sapphire and cherry lagtime2 in a well; venus, sapphire and cherry error code in a well. The semantics of these associations are that initial and final fluorescence, doubling time, lagtime2 and error code measurements were produced by the assay process and are present at the end of the process. Additionally, the relation has-target-origin was introduced to link a target and an organism of origin. We included this relation and other entities that are required to define semantic meaning of Eve data in a small ontology EVE that was specially designed to support the semantic data model of Eve's-screening assay results (http://disc.brunel.ac.uk/eve.). The node ‘DHFR target’ is linked via this relation to the host (*Homo sapiens*) and parasites. A target may be drug-resistant. This is expressed via the link sio:has-quality. The dataset is deposited at http://disc.brunel.ac.uk/eve-dataset.

To facilitate the reuse of the code, we have placed all the software: Eve low-level control software, QSAR software and active learning software on GitHub using the GNU General Public License v. 3 (https://github.com/RobotEve/RobotEve).

## 7. Discussion and conclusion

Eve's standardized assays could easily be engineered for other targets classes or target species (e.g. bacteria), for adjunctive targets (e.g. to drug import or efflux pumps) or for combinatory functions (e.g. to screen for drug synergies across multiple targets). In addition, the biological realism of the assays could be increased by the incorporation of multiple parasite targets within that same yeast cell, creating increasingly parasite-mimetic and human-mimetic cells. The assays could also be modified to be much faster—as using growth as the read-out limits the speed of executing the assay.

The economics of drug development are influenced by many factors [1–4] some technical (understanding how to intervene to treat a disease, the difficulty of achieving the intervention, etc.), others societal (safety standards, the drug pricing, etc.). Although the costs of drug discovery are substantial, they are relatively small compared with later stages in development. Such arguments tell against increased automation and standardization in drug discovery making much economic difference. However, they fail to take into account the ‘art of the soluble’ (Sir Peter Medawar). Preventing drug failures in late-stage development is an intrinsically very hard problem, as human biology is very complex. By contrast, we argue that a radical decrease in the cost and increase in the speed of drug discovery could be achieved by the full automation and standardization of procedures. By this, we mean a robotic system that once given a target could autonomously develop a standardized assay for that target, screen a compound library using that assay, confirm hit compounds and identify lead compounds through cycles of QSAR learning and testing. This could be achieved today: Eve's synthetic biology assays could be automated using existing technology, and chemical synthesis machines exist that could be integrated with Eve [11]. Such integration would achieve the goal of a robotic system that could autonomously generate hits for targets, and radically decrease the cost and increase the speed of drug discovery.

## Supplementary Material

williams_supplementary

## References

[1] Ioset JR Chang S 2011 Drugs for neglected diseases initiative model of drug development for neglected diseases: current status and future challenges. Future Med. Chem. 3, 1361–1371. (10.4155/fmc.11.102) 21879842

[2] Leslie M 2011 Drug developers finally take aim at a neglected disease. Science 333, 933–935. (10.1126/science.333.6045.933) 21852468

[3] Braun MM Farag-El-Massah S Xu K Coté TR 2010 Emergence of orphan drugs in the United States: a quantitative assessment of the first 25 years. Nat. Rev. Drug Disc. 9, 519–522. 10.1038/nrd3160 20531273

[4] Pammolli F Magazzini L Riccaboni M 2011 The productivity crisis in pharmaceutical R&D. Nat. Rev. Drug Disc. 10, 428–437. (10.1038/nrd3405) 21629293

[5] Gad SC 2005 Drug discovery handbook. New York, NY: Wiley.

[6] Martin YC 2010 Quantitative drug design: a critical introduction, 2nd edn Boca Raton, FL: CRC Press.

[7] Bernal JD 1969 Science in history, 4 vols. Harmondsworth, UK: Penguin.

[8] King RD Whelan KE Jones FM Reiser PGK Bryant CH Muggleton SH Kell DB Oliver SG 2004 Functional genomic hypothesis generation and experimentation by a robot scientist. Nature 427, 247–252. (10.1038/nature02236) 14724639

[9] King RD 2009 The automation of science. Science 324, 85–89. (10.1126/science.1165620) 19342587

[10] Sparkes A 2010 Towards robot scientists for autonomous scientific discovery. Autom. Exp. 2, 1–22. (10.1186/1759-4499-2-1) 20119518 PMC2813846

[11] Baumann M Baxendale IR Ley SV Martin RE Schneider J 2011 Synthesis of a drug-like focused library of trisubstituted pyrrolidines using integrated flow chemistry and batch methods. ACS Comb. Sci. 13, 405–413. (10.1021/co2000357) 21528880

[12] Rasmussen CE Williams CKI 2006 Gaussian processes for machine learning. Cambridge, MA: The MIT Press.

[13] De Grave K Ramon J De Raedt L 2008 Active learning for high throughput screening, pp. 185–196. Lecture Notes in Computer Science, vol. 5255 Berlin, Germany: Springer.

[14] O'Boyle NM Banck M James CA Morley C Vandermeersch T Hutchison GR 2011 Open Babel: an open chemical toolbox. J. Cheminform. 3, 33 (10.1186/1758-2946-3-33) 21982300 PMC3198950

[15] Cohn DA Ghahramani Z Jordan MI 1996 Active learning with statistical models. J. Artificial Intell. Res. 4, 129–145.

[16] Lounkine E 2012 Large-scale prediction and testing of drug activity on side-effect targets. Nature 486, 361–367. (10.1038/nature11159) 22722194 PMC3383642

[17] Vogel HG 2002 Drug discovery and evaluation: pharmacological assays. Berlin, Germany: Springer.

[18] Schneider G 2010 Virtual screening: an endless staircase? Nat. Rev. Drug Disc. 9, 273–276. (10.1038/nrd3139) 20357802

[19] Deutsch D 1985 Quantum theory, the Church–Turing principle and the universal quantum computer. Proc. R. Soc. Lond. A 400, 97–117. (10.1098/rspa.1985.0070)

[20] Benenson Y Gil B Ben-Dor U Adar R Shapiro E 2004 An autonomous molecular computer for logical control of gene expression. Nature 429, 423–429. (10.1038/nature02551) 15116117

[21] Smith AM Ammar R Nislow C Giaever G 2010 A survey of yeast genomic assays for drug and target discovery. Pharmacol. Ther. 127, 156–164. (10.1016/j.pharmthera.2010.04.012) 20546776 PMC2923554

[22] Bilsland E Pir P Gutteridge A Johns A King RD Oliver SG 2011 Functional expression of parasite drug targets and their human orthologs in yeast. PLoS Negl. Trop. Dis. 10, e1320 (10.1371/journal.pntd.0001320) PMC3186757 21991399

[23] Bilsland E 2013 Yeast-based automated high-throughput screens to identify antiparasitic lead compounds. Open Biol. 3, 120158 (10.1098/rsob.120158) 23446112 PMC3603448

[24] Chong CR Sullivan DJ 2007 New uses for old drugs. Nature 448, 645–646. (10.1038/448645a) 17687303

[25] Bizer C Heath T Berners-Lee T 2009 Linked data—the story so far. Intl. Semantic Web Info Syst. 5, 1–22.

[26] Zhang P Nicholson DE Bujnicki JM Su X Brendle JJ Ferdig M Kyle DE Milhous WK Chiang PK 2002 Angiogenesis inhibitors specific for methionine aminopeptidase 2 as drugs for malaria and leishmaniasis. J. Biomed. Sci. 9, 34–40. (10.1007/BF02256576) 11810023

[27] Chen X Xie S Bhat S Kumar N Shapiro TA Liu JO 2009 Fumagillin and fumarranol interact with *P. falciparum* methionine aminopeptidase 2 and inhibit malaria parasite growth *in vitro* and *in vivo*. Chem. Biol. 16, 193–201. (10.1016/j.chembiol.2009.01.006) 19246010

[28] Arico-Muendel C 2009 Antiparasitic activities of novel, orally available fumagillin analogs. Bioorg. Med. Chem. Lett. 19, 5128–5131. (10.1016/j.bmcl.2009.07.029) 19648008 PMC2745105

[29] Leartsakulpanich U Imwong M Pukrittayakamee S White NJ Snounou G Sirawaraporn W Yuthavong Y 2002 Molecular characterization of dihydrofolate reductase in relation to antifolate resistance in *Plasmodium vivax*. Mol. Biochem. Parasitol. 119, 63–73. (10.1016/S0166-6851(01)00402-9) 11755187

[30] Yuthavong Y 2012 Malarial dihydrofolate reductase as a paradigm for drug development against a resistance-compromised target. Proc. Natl Acad. Sci. USA 109, 16 823–16 828. (10.1073/pnas.1204556109) PMC3479511 23035243

[31] Besnard J 2012 Automated design of ligands to polypharmacological profiles. Nature 492, 215–219. (10.1038/nature11691) 23235874 PMC3653568

