# Supplementary material for: Cheaper faster drug development validated by the repositioning of drugs against neglected tropical diseases
Source: J R Soc Interface. 2015 Mar 6;12(104):20141289. doi: 10.1098/rsif.2014.1289 (PMC4345494; doi:10.1098/rsif.2014.1289)
Supplement: williams_supplementary [file rsif20141289supp1.pdf]

Williams *et al.* Supporting Online Material

1. **Standardised Analog Computer Assays**
   1.1 Yeast strains and plasmid constructs

2. **Library-screening assays using the Robot Scientist Eve**

3. **Compound Screening Software**
   3.1 Analysis of library-screening data
   3.2 Analysis of confirmation and intelligent screening data

4. **Automated QSAR Learning**
   4.1 Machine Learning
   4.2 Physically implementing active-learning
   4.3 Active-learning simulations
   4.4 Econometric modelling

5. **Ontological Description Used for Screening Results**

6. **Screening Results**

7. ***In vitro* Enzyme Assays**

8. **References**

1. Standardised Analog Computer Assays

1.1 Yeast strains and plasmid constructs

The list of strains constructed is given in Table S1. These are described in Bilsland *et al*. (*1*). Briefly, fluorescent plasmids were constructed by replacing the coding region of yEmRFP from yEpGAP-Cherry (*2*) with Venus or Sapphire (*3*) and replacing the *URA3* marker with *LEU2*. The strain expressing the drug-resistant *P.vivax* DHFR (^{PvR}dhfr) was constructed by mutating the following sites of the target enzyme: S58R, S117N and I173L. The plasmid was transformed into a yeast strain with a *dfr1*Δ*/DFR1 pdr5*Δ*/PDR5* BY4743 background. The strain was sporulated and *MAT*α haploids were selected for drug screens. The drug resistant ^{PfR}dhfr strain is a triple mutant for residues N51I, C59R & S108N, the required changes to the wild-type sequence having been made by site-directed mutagenesis.

These three fluorescent proteins have very distinct excitation and emission patterns: mCherry (ex 580nm; em 612nm), Sapphire (ex 405nm; em 510 nm) and Venus (ex 500nm; em 540 nm).

Strain name	Genotype	Plasmid
BY4743	*MAT**a**/MATα his3Δ1/his3Δ1 leu2Δ0/leu2Δ0 met15Δ0/MET15 LYS2/lys2Δ0 ura3Δ0/ura3Δ0*	
y^{Pf}DHFR_p	*dfr1Δ::Kan*MX *pdr5Δ::His*MX *his3Δ1 leu2Δ0 MET15 lys2Δ0 MATα*	pCM^{Pf}DHFR
y^{PfR}dhfr_p	*dfr1Δ::Kan*MX *pdr5Δ::His*MX *his3Δ1 leu2Δ0 MET15 lys2Δ0 MATα*	pCM^{PfR}dhfr
y^{Pv}DHFR_p	*dfr1Δ::Kan*MX *pdr5Δ::His*MX *his3Δ1 leu2Δ0 MET15 lys2Δ0 MATα*	pCM^{Pv}DHFR
y^{PvR}dhfr_p	*dfr1Δ::Kan*MX *pdr5Δ::His*MX *his3Δ1 leu2Δ0 MET15 lys2Δ0 MATα*	pCM^{PvR}dhfr
y^{Sm}DHFR_p	*dfr1Δ::Kan*MX *pdr5Δ::His*MX *his3Δ1 leu2Δ0 MET15 lys2Δ0 MATα*	pCM^{Sm}DHFR
y^{Tc}DHFR_p	*dfr1Δ::Kan*MX *pdr5Δ::His*MX *his3Δ1 leu2Δ0 MET15 lys2Δ0 MATα*	pCM^{Tc}DHFR
y^{Tb}DHFR_p	*dfr1Δ::Kan*MX *pdr5Δ::His*MX *his3Δ1 leu2Δ0 MET15 lys2Δ0 MATα*	pCM^{Tb}DHFR
y^{Lm}DHFR_p	*dfr1Δ::Kan*MX *pdr5Δ::His*MX *his3Δ1 leu2Δ0 MET15 lys2Δ0 MATα*	pCM^{Lm}DHFR
y^{Hs}DHFR_p	*dfr1Δ::Kan*MX *pdr5Δ::His*MX *his3Δ1 leu2Δ0 MET15 lys2Δ0 MATα*	pCM^{Hs}DHFR

Plasmid name	Features	Derived from
pCM188	CEN plasmid; TetO2 promoter, *URA3*	
pCM^{Pf}DHFR	^{Pf}DHFR, *URA3*	pCM188
pCM^{PfR}dhfr	Drug resistant ^{Pf}dhfr^{51I,59R,108N}, *URA3*	pCM188
pM^{Pv}DHFR	^{Pv}DHFR, *URA3*	pCM188
pCM^{PvR}dhfr	Drug resistant ^{Pv}dhfr^{58R,117N,172L}, *URA3*	pCM188
pCM^{Sm}DHFR	^{Sm}DHFR, *URA3*	pCM188
pCM^{Tb}DHFR	^{Tb}DHFR, *URA3*	pCM188
pCM^{Tc}DHFR	^{Tc}DHFR, *URA3*	pCM188
pCM^{Lm}DHFR	^{Lm}DHFR, *URA3*	pCM188
pCM^{Hs}DHFR	^{Hs}DHFR, *URA3*	pCM188
yEpVenus_LEU	2µ; TDH3-promoter-driven Venus, *LEU2*	yEpVenus_URA
yEpSapphire_LEU	2µ; TDH3-promoter-driven Sapphire (BFP), *LEU2*	yEpSapphire_URA
yEpCherry_LEU	2µ; TDH3-promoter-driven yEmRFP, *LEU2*	yEpGAP-Cherry

Table S1. Yeast strains and plasmids

The Yeast strains were cultured in YNB-glucose (0.68% yeast nitrogen base without amino acids, 2% ammonium sulphate, 2% glucose) with the relevant supplements for all assays.

2. Library-screening assays using the Robot Scientist Eve

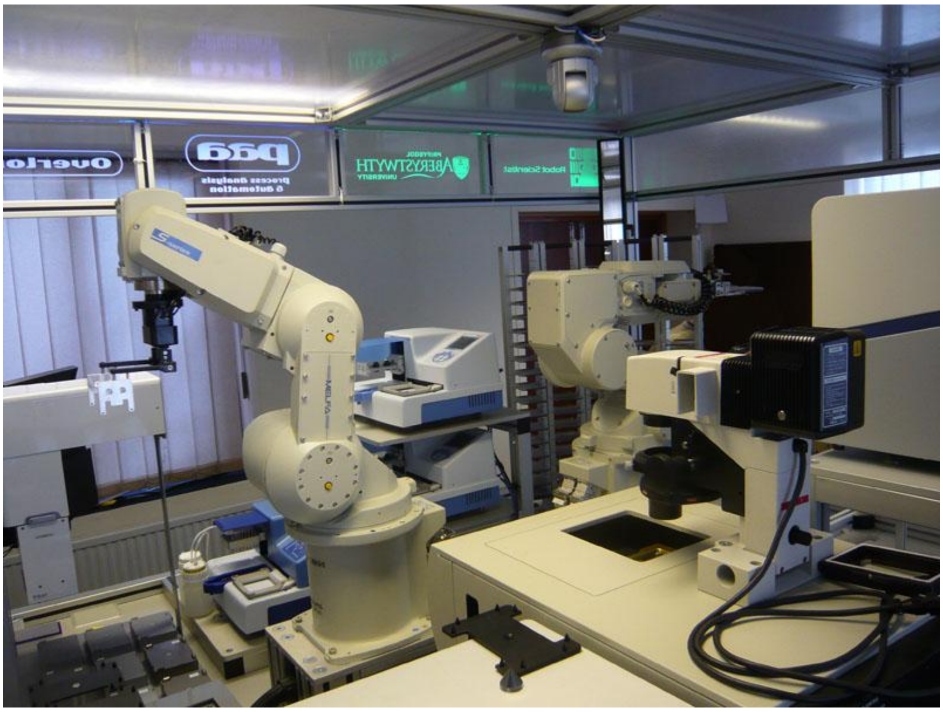

Fig. S1. The Robot Scientist, Eve, in action.

The initial plan of Eve was given in (*4*) (Fig. S1), and hardware and lov level software are described in the main text.

Establishment of the mixed cultures

Pre-cultures were grown to stationary phase and 1mL of each culture was inoculated into 100mL of fresh medium. Pools were incubated at 30°C, with shaking, for 4h to ensure exponential growth. Doxycycline (5μg/mL) was then added to the culture to reduce expression of the target enzyme (*1*). The culture was attached to a Thermo Combi multidrop within the Eve work cell. The culture was stirred continuously and maintained at 23°C during assay plate set up.

Screening and hit confirmation, using the Robot Scientist Eve

Screens were performed by the Robot Scientist Eve using mixed cultures described above and either the ~1,600 FDA-and foreign-approved drugs from the Johns Hopkins University Clinical Compound Library or the 14,400 compound Maybridge Hitfinder library. Strains were grown in competition in the presence of a library compound, as discussed above, and the relative growth rates used to estimate the activity of the drug against the parasite target.

A typical Maybridge library screen consists of a set of 45 384-well plates, each well containing a pool of three yeast strains harboring either a parasite drug target or its human counterpart. Different Maybridge compounds were added to each of 320 wells on each plate. 20 of the remaining wells were used for positive controls (pyrimethamine, trimethoprim, methotrexate, raltitrexed monohydrate, pemetrexed disodium), and 44 for negative controls, with strains growing in an equivalent concentration of DMSO. Each well was inoculated with 50 μL of the pooled yeast

culture (final compound concentration of 10 μM). Plates were incubated for 40 hours, and fluorescence measurements taken every 90 minutes.

For hit confirmation assays, each plate consisted of eight replicates of eight different compounds, at six different concentrations (0, 1, 2.5, 5, 10, 20 μM), and 64 negative control wells.

3. Compound Screening Software

3.1 Analysis of library-screening data

Growth curve parameter derivation

Growth curves were fit to the time course, and growth parameters A-P (Fig. S2) derived (*5*, *6*).

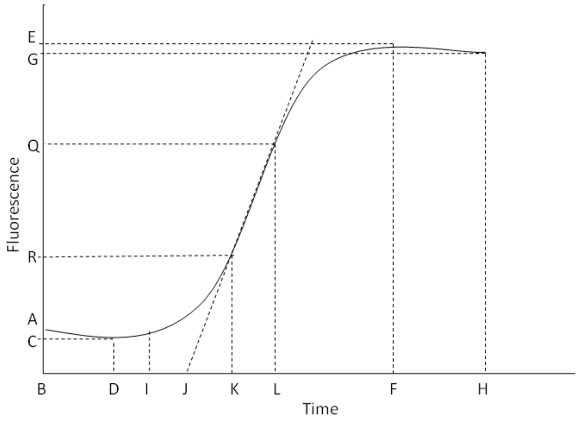

A	startfluorescence	I	lagtime
B	startfluorescencetime	J	lagtime2
C	minfluorescence	K	startlinear
D	minfluorescencetime	L	endlinear
E	maxfluorescence	M	linearslope = (Q - R)/(L - K)
F	maxfluorescencetime	N	doubletime = 1/M
G	endfluorescence	O	durlinear = L - K
H	endfluorescencetime	P	snratio (signal/noise ratio)

Figure S2. Typical growth curve, and growth parameters extracted from the curve.

Reproducibility of growth curve parameters

The variation of doubling time (DT) amongst the negative controls within a plate was used to estimate in-plate repeatability, and between-plate reproducibility was estimated using DT of both the negative and positive controls. Example data from screen TS3 (Hs, Pv, PfR) is shown in Table S2.

Batch	Plates	mcherry DT HsDHFR		sapphire DT PvDHFR		venus DT PfRdhfr		Sample size
		Mean	Variance	Mean	Var	Mean	Var	
1	2125-2132	2.05	0.0046	3.64	0.0062	2.90	0.0037	415
2	2133-2140	2.04	0.0062	3.52	0.0042	2.86	0.0024	514
3	2141-2148	2.04	0.0077	3.15	0.0032	2.87	0.0035	514
5	2158-2165	2.05	0.0055	3.39	0.0038	3.02	0.0036	440
6	2170-2174	2.09	0.0037	3.56	0.0035	2.91	0.0024	298
Average		2.05	0.0061	3.44	0.0358	2.91	0.0065	2181

Table S2. Demonstration of reproducibility between Eve screens

DT was then used to identify compounds causing significantly reduced growth (> two standard deviations) relative to the negative controls. For screen TS3, approximately 600 compounds were identified as potential hits in this way. These were then classified according to the remaining growth parameters (Fig. S2), as (a) autofluorescent; (b) cross-inhibited and (c) hit compounds (Fig. S3) using the following criteria:

(a) Autofluorescent compounds – fluorescence of the drug itself interfering with the growth assay

1. High fluorescence at one or more wavelengths throughout the run
2. High initial fluorescence (>8% higher than the negative control value) at one or more wavelengths

(b) Compounds with evidence of cross-inhibition

1. Low intensity fluorescence for all strains
2. Low growth for all strains
3. Long lag time for all strains

Note: Compounds with these properties can either be toxic to yeast (in that case, they would appear as toxic in all of the screens, irrespective of the target), problem wells (technical/experimental error), or compounds that inhibit yeast growth when either the parasite enzyme or its human ortholog are expressed.

(c) Hit compounds – candidate anti-parasitic drugs

1. Reduced yield for a parasite strain relative to the human strain
2. Reduced growth rate for a parasite strain relative to the human strain

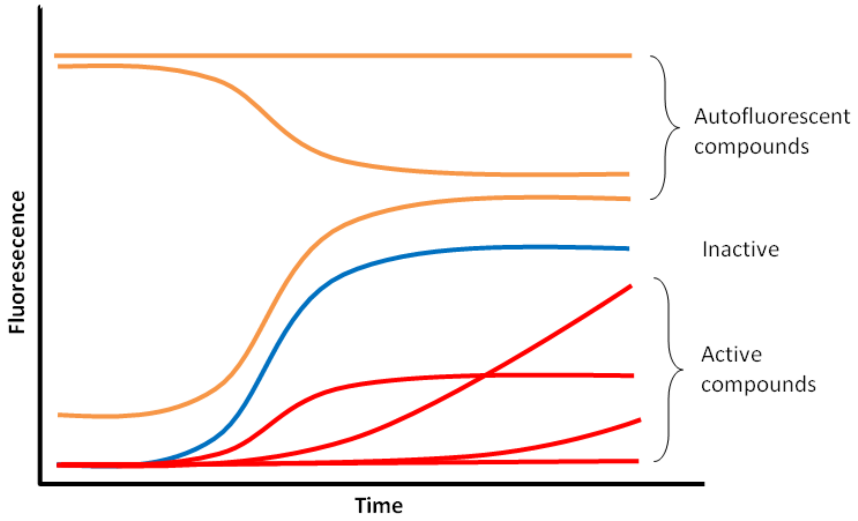

Figure S3. Typical forms of growth curves observed in Eve drug screens, and the classification of compounds based on the curve properties.

Derivation of Rules for deciding library-screening hits

325 of these initial candidate hit compounds, based on abnormal DT, were categorised manually, resulting in:

- 83 possibly toxic compounds
- 16 autofluorescent compounds
- 57 strong and 64 weak hits against PvDHFR
- 9 weak hits against PfRDHFR
- 67 inactive compounds

Based on this primary data, Weka 3.6.2, C4,5 (J48) decision trees (*7, 8*) were used to determine an optimal set of rules for automatically classifying growth curves.

The rules defined by the decision trees were based on a subset of the growth parameters (Fig. S2). These were: the strain 'yield ratio' - the change in fluorescence when grown in the presence of a drug, relative to the negative control; the initial strain fluorescence (relative to the change in fluorescence of the negative control); doubling time relative to the negative control; and lagtime2 relative to the negative control. The combined growth of all strains within a well was also employed in the decision trees to distinguish cross-inhibited compounds.

The decision tree process produced very similar rules when by considering each of the Hs, Pv and PfR datasets individually. These were combined into a generalised set of rules which were used in all subsequent screens (Table S3).

Potential hit	strain_yield_ratio < 0.8	Low yield (<80% relative to the negative control)
	strain_yield_ratio > 0.8<br>strain_DT > 1.5	Low growth rate (>50% slower growth than the negative control)
	strain_yield_ratio > 0.8<br>strain_lagtime2 < -4	Long lag time (>4 hours longer than the negative control)

Table S3. Machine learning rules derived to identify potential hit compounds with the Eve mass screen.

3.2 Analysis and confirmation of intelligent screening data

After performing a mass screen and classifying compounds according to the rules defined above, confirmation screens were performed on the potential hit compounds. The same protocols were employed during the intelligent screening process. Machine learning rules for the cherry-picked screens were developed using Weka (J48 decision trees) as above. Rules were validated by comparing machine-learning classifications with inspection of the growth curves, which resulted in near-complete agreement for all screens.

Category	Rule	Interpretation
Venus hit<br>(40 curves)	ven_hit ≤ 3<br>[23 inactive/4 weak/2 active]<br>ven_hit > 3<br>[23 active/11 inactive]<br>46/63 = 73% prediction success	If more than three of the 40 individual venus curves are hits, then the compound is active against the venus-labelled target
Sapphire hit<br>(40 curves)	sap_hit ≤ 3<br>[4 inactive/1 active]<br>3 < sap_hit < 5<br>[2 weak]<br>sap_hit > 5<br>[active 53/inactive 3]<br>58/63 = 90% prediction success	If more than five of the 40 individual sapphire curves are hits, then the compound is active against the sapphire-labelled target.
Cross inhibition<br>(40 curves)	che_hit ≤ 4<br>[48 inactive/2 active]<br>che_hit > 4<br>[11 active/2 inactive]<br>59/63 = 92% prediction success	If more than four of the 40 individual cherry curves are hits, then the compound is active against the cherry-labelled (Hs) target, and is classed as possibly cross-inhibited.

Table S4. Machine learning rules derived to identify hit and cross-inhibited compounds within Eve cherry-picking screens.

4. Automated QSAR Learning

4.1 Machine Learning

To form QSAR hypotheses Eve uses a Gaussian process model (*9*), with the molecules represented using binary fingerprints representing all linear paths of up to seven atoms, computed using Open Babel (*10*). The inputs for learning were OpenBabel FP2 fingerprints (using the 0/7 configuration) for compound SMILES codes (training set and unknowns), and yield ratio differences between target and human strains, e.g (strain_yield_ratio$_{HsDHFR}$ – strain_yield_ratio$_{PvDHFR}$).

4.2 Implementing Active Learning loops

The data analysis and subsequent rules were explained above and were combined with the following to enable an autonomous process:

- a method to select ‘hit’ compounds (either by active learning or by selection for a confirmation screen based on the decision tree rules)
- a method to combine the confirmation/intelligent screen assays complex cherry-pick results with the simpler mass screen data, to build the information source for the active learning feedback loop (Fig. S4).

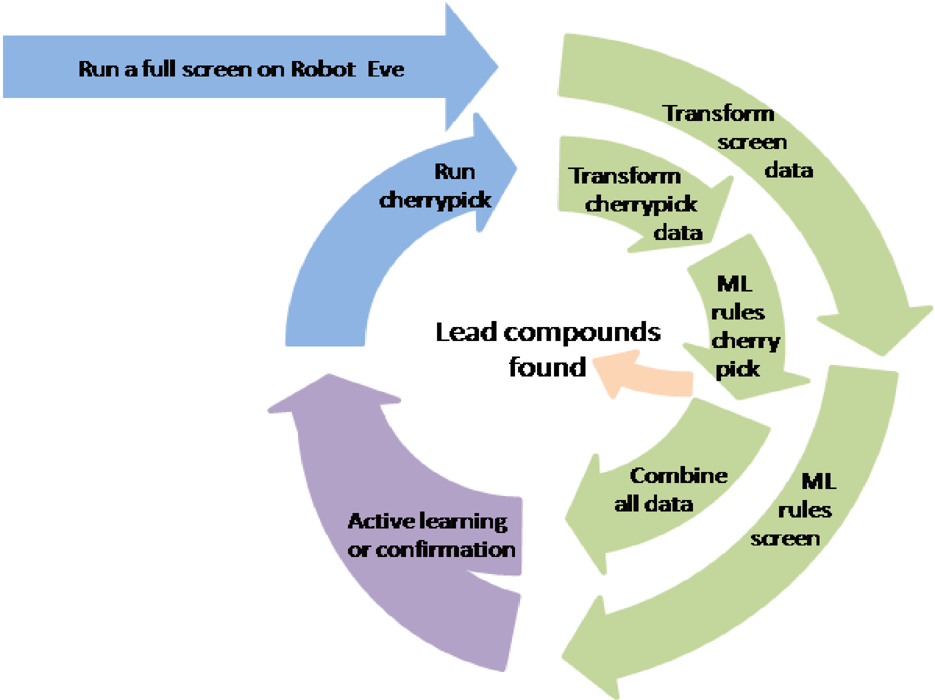

Figure S4. Eve’s strategy for improving the efficiency of library screening by the application of machine learning.

The first full experimental tests of the active learning loop were conducted by splitting the screen data set for TS6 (comprising the heterologous DHFR yeast strains for *Plasmodium falciparum and P.vivax*, and that of humans), and using 4800 compounds as a training set. The yield ratios of the HsDHFR and PvDHFR strains were passed to the selection algorithm, together with fingerprints of the remaining 9600 compounds. The results from the first cherry-picking round (n=96; 12 plates of 8 compounds per plate; 8 replicates of 6 concentrations) were then added to the original data set, and a second cherry-picking round conducted. To examine different approaches to the problem of combining cherry-picking and mass screening data,

seven versions of weighted cherry-picking data ($strain\_yield\_ratio_{HsDHFR}$ – $strain\_yield\_ratio_{PvDHFR}$) were tested in the second round:

i. All replicates for each compound
ii. All replicates multiplied by 10/conc
iii. All replicates multiplied by log(10/conc)
iv. Mean of replicates at each concentration for each compound
v. Mean of replicates multiplied by 10/conc
vi. Mean of replicates multiplied by log(10/conc)
vii. No additional data (i.e. the next best 96 compounds under loop1 conditions)

When the ML rules were applied to TS6, 282/14099 compounds (2.0%) were identified as active against the Pv strain. The curves for the cherry-picking data were empirically categorised, and evaluated against the weighting options, with the log-weighted option (vi) being deemed optimal.

	Loop		Weighting options, loop 2						
	1	2	i	ii	iii	iv	v	vi	vii
Compounds	96	188	96	96	96	96	96	96	96
Hits	3	9	7	6	8	6	7	9	1
Hit/cross-inhibited	-	3	3	3	3	3	3	3	2
Weak activity	3	16	8	8	10	8	8	7	6
Weak/ cross-inhibited	1	-	-	-	-	-	-	-	-
Possibly cross-inhibited	-	8	2	6	3	2	6	2	3
No activity	87	160	76	73	72	77	72	75	84

Table S5. Methods for integrating cherry-picking screen data into Eve's active learning algorithm.

4.3 Active Learning simulations

The active learning loop was run through three iterations; an initial set of 4800 compounds was screened (single iteration, 10 µM), and three loops of 96 cherry-picked compounds (8 replicates, at a range of concentrations) were selected. The mean log.-weighted cherry-picking data is cycled back into the training set. In addition to physical active-learning screens, simulations were performed, using the empirical data for each compound in the whole-library screen dataset to simulate log-weighted cherry-picking scores for the compound. Experiment and simulation are compared in Table S6.

Loop	Hits			Weak activity		
	Experiment	Both	Simulation	Experiment	Both	Simulation
1	5	5	5	3	3	5
2	11	10	11	4	4	4
3	4	2	8	0	0	3

Table S6. Active compounds in each 96 compound Active Learning loop

4.4 Econometric modelling

The model

To determine the utility of Eve for drug discovery – i.e. the range of conditions for which using a Robot Scientist to guide candidate compound selection is economically advantageous compared with performing a standard whole-library screen – we developed the econometric model presented in Figure 2 in the main text (and below).

$$\Delta\, Utility\ of\ Eve = \sum_{1}^{Nm}(Tm + Cm) + \sum_{1}^{Nx}(Tc + Cc - Uh) + \sum_{1}^{Ne}(Tm - Tc + Cm - Cc)$$

Nm	-	Number of compounds not assayed by Eve
Tm	-	Cost of the time to screen a compound using the mass screening assay
Cm	-	Cost of the loss of a compound in the mass screening assay
Nx	-	Number of hits missed by Eve
Tc	-	Cost of the time to screen a compound using a cherry-picking (confirmation or intelligent) assay
Cc	-	Cost of the loss of a compound in a cherry-picking assay
Uh	-	Utility of a hit
Ne	-	Number of compounds assayed by Eve

Figure S5: Econometric model for the differential advantage of intelligent screening

The net utility is made up of three components: the cost saving due to not screening *Nm* compounds which, based on the QSAR learning, are unlikely to be hits, minus the opportunity cost due to not finding any hits (*Nx*) that might be present in this unscreened set ($Uh >> Tc + Cc$), minus the cumulative cost of the number of active learning cycles performed [the cost of cherry-picking *Ne* compounds, where $(Tc + Cc) > (Tm + Cm)$].

Active k-optimisation strategy

One of the major goals for Eve's data analysis was to build algorithms to predict active compounds. The array of information contained in assay data includes raw data for yeast target growth profiles, labelled classifications for activity, toxicity etc., and structural representations of the compounds.

The prototype method for this work is based on the *active k-optimisation* strategy (*11*). This strategy is introduced for machine learning as a way of finding and ranking the *k* best alternatives for evaluation, using Gaussian process to provide a mechanism for developing this model. The process finds more than one target (other than the optimal solution) to take into the next step of HTE. The work is based on having a finite library of examples, of which the results from the known ones can be used to pick the best unknowns for evaluation.

In this particular approach, the goal is to pick targets that have the best chance of success (the **maximum predicted** strategy) for comparison to several other existing approaches (*6, 12, 13*). The lower confidence bound criterion (**optimistic**) (*14*), selecting the sample with the highest probability of improving the current solution

(**most probable improvement, MPI**) and efficient global optimisation (EGO) to give **maximum expected improvement**.

Specific application of the *active k-optimisation strategy* to the drug screening process is provided in (*15*); this describes the analysis of the NCI60 dataset (US National Cancer Institute, 60 anticancer drug screen). The full techniques upon which this strategy is based are given in (*9*).

Cherry-picking simulations using active k-optimisation

The inputs for the prototype AL method are OpenBabel FP2 fingerprints (using the 0/7 configuration) (*16*, *17*) for compound SMILES codes (*18*, *19*) (training set and unknowns), and simple growth differences between target and human strains, i.e.

$$(strain\_yield\_ratio_{Hs} - strain\_yield\_ratio_{target})$$

The method is designed to identify the next best compound to test from a given library, whilst avoiding selection of a compound very similar to any in the training set; it is expected to balance exploration of the full chemical space of the library, with exploitation of areas most likely to contain active examples.

Econometric modelling using active k-optimisation simulation data

The *active k-optimisation* AL algorithm was applied to the seed input data and the unknown compound SMILES codes; simulated learning curves were produced for each parasite strain using the proxy confirmation data (see section 4.2). The progression of these learning curves was then compared to the base case of a linear progression throughout the screen in accordance to the utility equation (fig. S5). For each 96-compound loop, the number of proxy confirmed hits and compounds screened to date (*Ne*) were applied to the utility equation, together with the fixed utility and cost terms. An example of the resultant 2D plot for the PvDHFR strain is shown in fig. S6.

Table S7: Simulation data for the TS3 PvDHFR target - number of hit compounds found with progression of the loop count.

	Compounds	Screen hits
Full screen	14386	316
Seed	958	25
Unknowns	13428	291

No. of loops	No. of screen hits in simulation loops						
	n=0	n=10	n=20	n=30	n=40	n=50	n=60
n+1	8	53	106	133	162	190	204
n+2	12	60	109	137	163	192	206
n+3	17	67	111	141	167	194	208
n+4	23	73	112	142	169	196	210
n+5	29	79	116	144	174	198	214
n+6	30	86	117	146	179	199	216
n+7	36	90	118	148	182	200	217
n+8	39	95	123	152	184	201	218
n+9	44	98	125	154	186	202	221
n+10	47	104	127	157	189	203	223

No. of loops	No. of screen hits						
	n=70	n=80	n=90	n=100	n=110	n=120	n=130
n+1	226	244	260	279	291		
n+2	228	247	263	280			
n+3	229	250	264	281			
n+4	231	251	268	283			
n+5	233	252	271	284			
n+6	235	253	273	285			
n+7	238	254	274	286			
n+8	240	255	275	288			
n+9	242	256	277	289			
n+10	243	259	278	290			

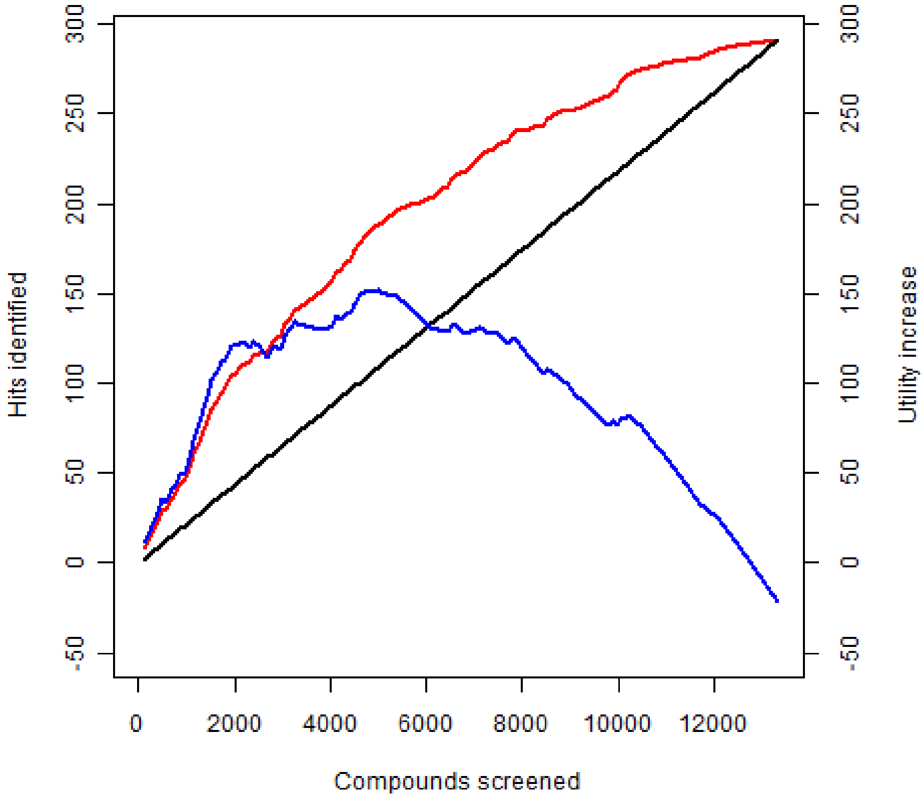

Figure S6: Hits found in TS3 PvDHFR simulation (red), base case (black), resultant econometric utility model (blue).

The cost of a cycle of ML includes both the time and cost of the computing power, and the cost of testing a 96-compound batch in the cherry-picking assay. Based on the *active k-optimisation* simulated cherry-picking cycles, these terms were also calculated for examples of each parasite target (figure S7).

A utility landscape was also constructed for the TS3 PvDHFR parasite data across a range of ML efficiencies and drug economic values (figure 2c); the value of a hit compound for this study ranged from $3K to $25K, based on a broad estimate of the number of hits required to give sufficient drug-like lead compounds to commence lead optimisation studies. Variation in the time-cost ratio comparing mass to intelligent screening (*Tc/Tm*) was studied, and utility versus cost of compound loss during cherry-picking (*Uh/Cc*) was also evaluated.

Screen	DHFR Target		DHFR Target	
TS3	Pv	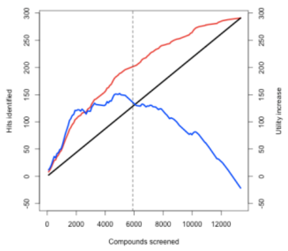	PfR	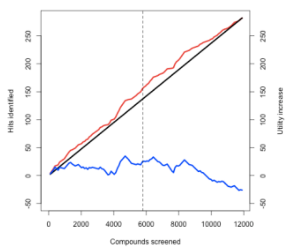
TS4	Tb	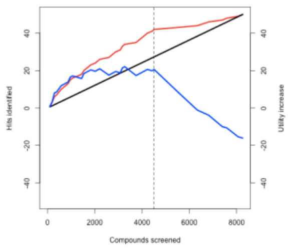	Sm	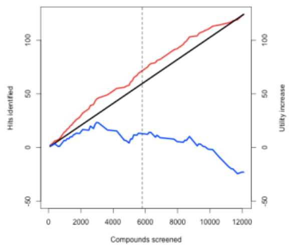
TS5	PfR	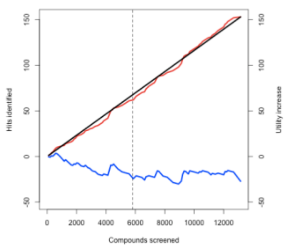	Tc	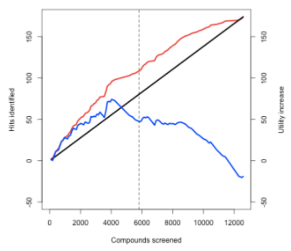
TS6	Pv	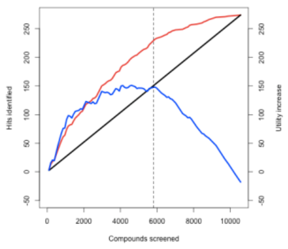	Pf	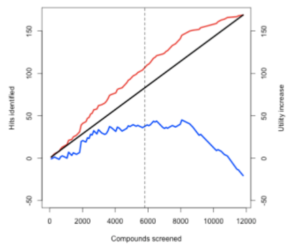
TS7	Lm	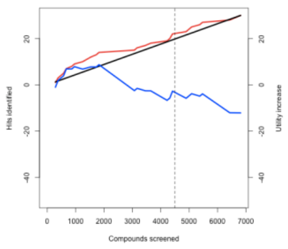	PvR	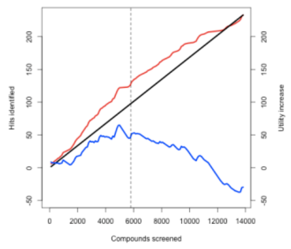

Figure S7: Simulations of intelligent screening for each DHFR target.

5. Ontological Description of the Data

Tables S8 and S9 contain the list of the relations, classes, and their URIs used for the semantic model shown at Fig. S8. The following semantic resources have been used to define the semantic meaning of Eve's screening assay results dataset:

- RO (the Relation Ontology) (http://obofoundry.org/ro)
- SIO (Semanticscience Integrated Ontology) (semanticscience.org/ontology/sio.owl)
- BFO (the Basic Formal Ontology) (www.ifomis.org/bfo/)
- PATO (Phenotypic Quality Ontology) (http://obofoundry.org/wiki/index.php/PATO:Main_Page)
- ChEBI (Chemical Entities of Biological Interest) (http://www.ebi.ac.uk/chebi)
- GO (The Gene Ontology) (http://www.geneontology.org/)
- PPI (Protein Protein Interactions) (http://purl.obolibrary.org/obo/mi.owl),
- OBI (The Ontology for Biomedical Investigations) (obi-ontology.org/)
- NCI Thesaurus (http://purl.bioontology.org/ontology/NCIt)
- NCBI (http://www.ncbi.nlm.nih.gov/Taxonomy),
- IDOMAL (Malaria Ontology) (http://purl.org/obo/owl/IDOMAL)

I	**Relation**	**Source**	**Semantic meaning**	**URI**
1.	is-a	rdf:-type	If X is-a Y, then all instances of the class X are instances of the class Y	http://www.w3.org/1999/02/22-rdf-syntax-ns
2.	has-input	RO	An entity X participates in a process Y and X is present at the beginning of the process Y.	http://purl.obolibrary.org/obo/RO_0002233
3.	has-output	RO	An entity X participates in a process Y and X is present at the end of the process Y.	http://purl.obolibrary.org/obo/RO_0002234
4.	has-agent	RO	Has participant Y if and only if X realizes some active role that inheres in Y	http:/purl.obolibrary.org/obo/RO_0002218
5.	has-part	BFO	For individual entities S and O, it holds that 'S has part O', if and only if 1) S and O are within the same mereologically dimensioned area (like space, time, space-time, or any ordered space including abstract space like in information entities), 2) the space occupied by O, is part of the space occupied by S.	http://purl.obolibrary.org/obo/BFO_0000051
6.	has-quality	SIO	A relation between an entity and the quality that it bears.	http://semanticscience.org/ontology/sio-core.owl#has-quality
7.	has-identifier	SIO	Has unique identifier is an inverse functional relation between an entity and an identifier that uniquely identifies it.	http://semanticscience.org/ontology/sio-core.owl#has-identifier
8.	has-target-origin	EVE	A relation between a target and an organism the target has been taken from	http://disc.brunel.ac.uk/eve#has-target-origin
9.	SameAS	OWL	A relation between equivalent entities	http://www.w3.org/2002/07/owl

Table S8. The relations used in the semantic model of Eve dataset.

	Class	Source	Semantic meaning	URI
1.	assay-triple-screen	EVE	A planned process with the objective to produce information about a compound	http://disc.brunel.ac.uk/eve#assay-triple-screen
2.	Compound-name	EVE	A commonly used name of a chemical entity that plays a role of compound in an assay triple screen	http://disc.brunel.ac.uk/eve# compound-name
3.	concentration	PATO	Quality of related physical entities inhering in a substance by virtue of the amount of the bearer's there is mixed with another substance.	http://purl.org/obo/owl/PATO#PATO_0000033
4.	maybridge-hit-finder-id		A unique identifier of a compound in the Maybridge library of compounds	http://disc.brunel.ac.uk/eve#maybridge-hit-finder-id
5.	initial-fluorescence	EVE	A relative measurement of the light emission at a particular wavelength by a given fluorophore at the beginning of an assay.	http://disc.brunel.ac.uk/eve# initial-fluorescence
6.	final-fluorescence	EVE	A relative measurement of the light emission at a particular wavelength by a given fluorophore at the end of an assay.	http://disc.brunel.ac.uk/eve# final-fluorescence
7.	doubling-time	EVE	The shortest time interval for the relative fluorescence at a particular wavelength to double	http://disc.brunel.ac.uk/eve# doubling-time
8.	lagtime2	EVE	A measurement of a time period until the start of an exponential growth of the relative fluorescence at each wavelength.	http://disc.brunel.ac.uk/eve# lagtime2
9.	error-code	EVE	A code that is generated by the robot Eve by the end of an assay triple screen. The code has value 0 if there were no errors, and 1 if there was an error.	http://disc.brunel.ac.uk/eve# error-code
10.	compound	EVE	A chemical entity that plays a role of compound in an assay triple screen and is represented by a SMILES code. The entity is equivalent to the class ChEBI: chemical-entity	http://disc.brunel.ac.uk/eve#smiles
11.	chemical-entity	ChEBI	A chemical entity is a physical entity of interest in chemistry including molecular entities, parts thereof, and chemical substances.	http://purl.obolibrary.org/obo/CHEBI_24431
12.	plate	EVE	A microtiter plate that is used in an assay triple screen	http://disc.brunel.ac.uk/eve#plate
13.	well	EVE	A part of a plate that plays a role of a container for participants of an assay, e.g. a compound, yeast cells, growth media A well is identified by a raw (a letter, A-P) and a column (a number, 1-24)	http://disc.brunel.ac.uk/eve#well
14.	synthetic-yeast-strain	EVE	A yeast strain specifically engineered for an assay	http://disc.brunel.ac.uk/eve#synthetic-yeast-strain
15.	DHFR-target	EVE	A target that has dihydrofolate reductase (GO:0004146) activity	http://disc.brunel.ac.uk/eve#dhfr-target
16.	drug-resistance	IDO-MAL	A protective mutation that mitigates the damaging effects of a drug.	http://purl.obolibrary.org/obo/IDOMAL_0000027
17.	fluorophore	PPI	A fluorophore is a component of a molecule which causes a molecule to be fluorescent. It is a functional group in a molecule which will absorb energy of a specific wavelength and re-emit energy at a different (but equally specific) wavelength. The amount and wavelength of the emitted energy depend on both the fluorophore and the chemical environment of the fluorophore.	http://purl.obolibrary.org/obo/MI_0856
18.	mcherry-fluorophore	EVE	A monomeric fluorescent construct which absorbs at 587 nm and emits at 610 nm.	http://disc.brunel.ac.uk/eve#mcherry-fluorophore
19.	sapphire-fluorophore	EVE	A monomeric fluorescent construct which absorbs at 399 nm and emits at 511 nm.	http://disc.brunel.ac.uk/eve#sapphire-fluorophore
20.	venus-fluorophore	EVE	A monomeric fluorescent construct which absorbs at 515 nm and emits at 528 nm.	http://disc.brunel.ac.uk/eve#venus-fluorophore
21.	organism	OBI	A material entity that is an individual living system, such as animal, plant, bacteria or virus, that is capable of replicating or reproducing, growth and maintenance in the right environment.	http://purl.obolibrary.org/obo/OBI_0100026

22.	human	NCI The-saurus	The bipedal primate mammal, Homo sapiens; belonging to man or mankind; pertaining to man or to the race of man; use of man as experimental subject or unit of analysis in research.	http://ncicb.nci.nih.gov/xml/owl/EVS/Thesaurus.owl#Human
23.	*Plasmodium vivax*	NCBI	An organism, malaria parasite *P. vivax*	Taxonomy ID: 5855
24.	*Plasmodium falciparum*	NCBI	An organism, malaria parasite *P. falciparum*	Taxonomy ID: 5833

Table S9. The classes used in the semantic model of Eve dataset.

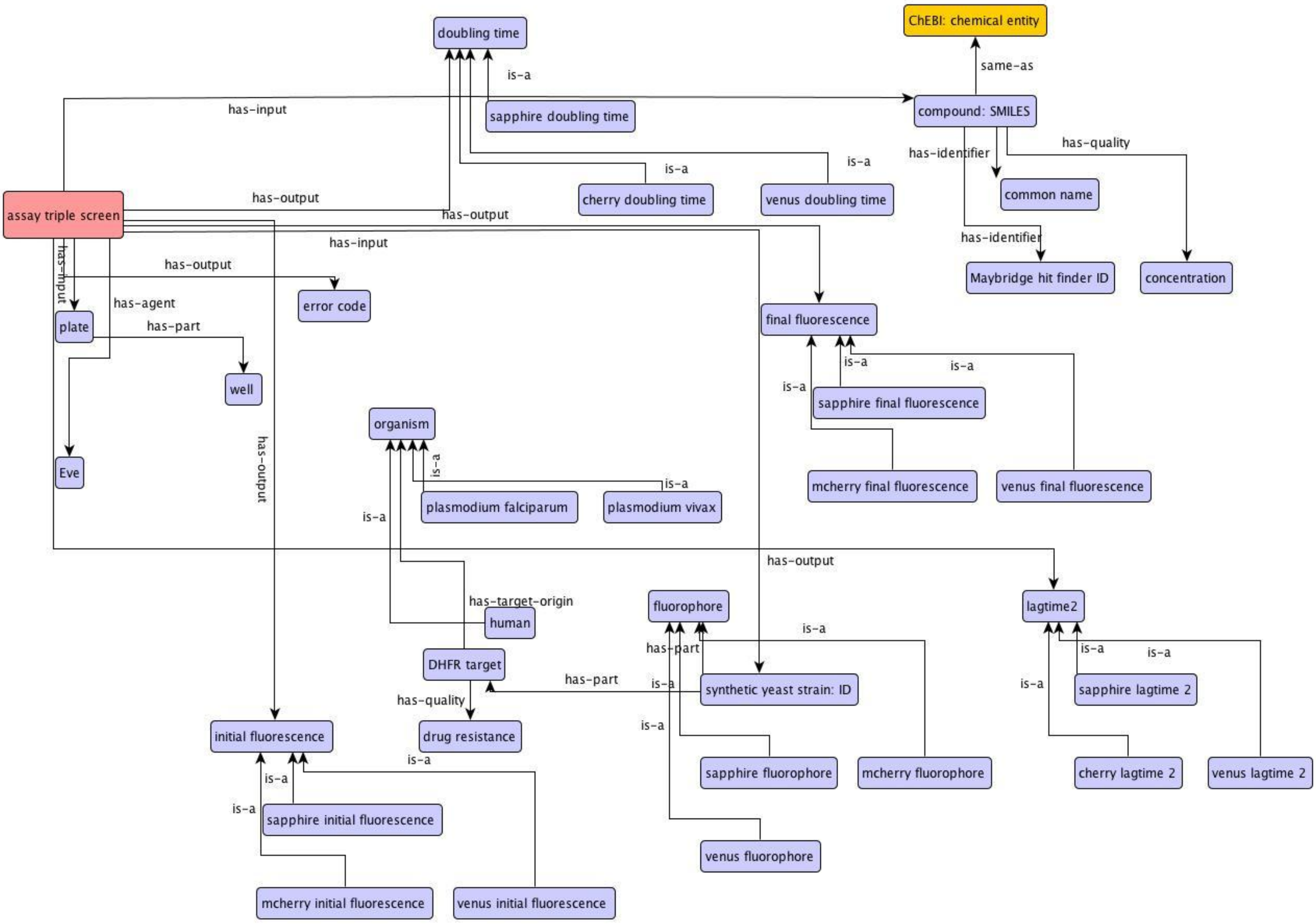

Fig. S8. A semantic data model of Eve dataset (simplified)

6. Screening Results

Table S10. DHFR TS3 assay, JHCCL compounds

	Eve ID	JHCCL ID	HsDHFR	PvDHFR	PfRdhfr	Pv active	PfR active	Evidence of cross inhibition
TS3_63_7	20110	SM_JHU-520	11	12	16	Yes	Yes	Possibly
TS3_63_7	20168	SM_JHU-904	8	8	22	Yes	Yes	Possibly
TS3_63_2	20168	SM_JHU-904	16	0	15		Yes	Possibly
TS3_63_3	20248	SM_JHU-1305	10	16	36	Yes	Yes	Possibly
TS3_63_7	20248	SM_JHU-1305	0	12	16	Yes	Yes	No
TS3_63_2	20414	SM_JHU-2095	8	32	1	Yes		No
TS3_63_7	20414	SM_JHU-2095	23	25	22	Yes	Yes	Possibly
TS3_63_2	20484	SM_JHU-2524	13	58	46	Yes	Yes	Possibly
TS3_63_2	21463	SM_JHU-9003	0	24	0	Yes		No

Table S11. DHFR TS4 assay, JHCCL compounds

	Eve ID	JHCCL ID	HsDHFR	TbDHFR	Sm DHFR	Tb active	Sm active	Evidence of cross inhibition
TS4_64_6	20248	SM_JHU-1305	8	8	24	Yes	Yes	Possibly
TS4_64_4	20516	SM_JHU-2766	0	4	12		Yes	No
TS4_64_4	20561	SM_JHU-3038	26	22	25	Yes	Yes	Possibly

Table S12. DHFR TS5 assay, JHCCL compounds

	Eve ID	JHCCL ID	PfRdhfr	HsDHFR	TcDHFR	PfR active	Tc active	Evidence of cross inhibition
TS5_71_2	20110	SM_JHU-520	21	33	40	Yes	Yes	Possibly
TS5_71_5	20110	SM_JHU-520	11	17	18	Yes	Yes	Possibly
TS5_71_6	20110	SM_JHU-520	12	6	9		Yes	No
TS5_71_2	20168	SM_JHU-904	4	25	50		Yes	Possibly
TS5_71_5	20168	SM_JHU-904	7	22	26		Yes	Possibly
TS5_71_6	20168	SM_JHU-904	2	14	22		Yes	Possibly
TS5_71_3	20248	SM_JHU-1305	8	8	8	Yes	Yes	Possibly
TS5_71_5	20248	SM_JHU-1305	8	10	16	Yes	Yes	Possibly
TS5_71_6	20248	SM_JHU-1305	10	16	20	Yes	Yes	Possibly
TS5_71_6	20414	SM_JHU-2095	14	21	19	Yes	Yes	Possibly
TS5_71_2	20427	SM_JHU-2151	56	59	64	Yes	Yes	Possibly
TS5_71_2	20561	SM_JHU-3038	16	12	18	Yes	Yes	Possibly

Table S13. DHFR TS6 assay, JHCCL compounds

	Eve ID	JHCCL ID	HsDHFR	PvDHFR	PfDHFR	Pv active	Pf active	Evidence of cross inhibition
TS6_77_11	20110	SM_JHU-520	17	11	14	Yes	Yes	Possibly
TS6_77_14	20110	SM_JHU-520	16	13	16	Yes	Yes	Possibly
TS6_c'pick	20141	SM_JHU-715	0	48	0	Yes		No
TS6_77_11	20168	SM_JHU-904	12	14	26	Yes	Yes	Possibly
TS6_77_16	20168	SM_JHU-904	14	13	18	Yes	Yes	Possibly
TS6_77_14	20168	SM_JHU-904	4	3	18		Yes	No
TS6_c'pick	20245	SM_JHU-1293	0	40	0	Yes		No
TS6_c'pick	20248	SM_JHU-1305	12	44	50	Yes	Yes	Possibly
TS6_77_11	20248	SM_JHU-1305	8	16	16	Yes	Yes	Possibly
TS6_77_14	20248	SM_JHU-1305	0	12	12	Yes	Yes	No
TS6_c'pick	20414	SM_JHU-2095	6	30	8	Yes		No
TS6_77_14	20414	SM_JHU-2095	20	24	24	Yes	Yes	Possibly
TS6_77_16	20414	SM_JHU-2095	22	24	21	Yes	Yes	Possibly
TS6_c'pick	20484	SM_JHU-2524	16	48	46	Yes	Yes	Possibly
TS6_c'pick	20516	SM_JHU-2766	10	20	16	Yes	Yes	Possibly
TS6_c'pick	20940	SM_JHU-5529	0	44	0	Yes		No
TS6_c'pick	21389	SM_JHU-8509	0	80	80	Yes	Yes	No
TS6_c'pick	21438	SM_JHU-8859	0	10	12	Yes	Yes	No
TS6_c'pick	21463	SM_JHU-9003	0	12	0	Yes		No
TS6_c'pick	21625	SM_JHU-10251	0	38	0	Yes		No
TS6_77_16	21767	SM_JHU-12096	7	11	11	Yes	Yes	No
TS6_c'pick	21767	SM_JHU-12096	8	9	12		Yes	No
TS6_77_14	21919		0	10	0	Yes		No

Table S14. DHFR TS7 assay, JHCCL compounds

	Eve ID	JHCCL ID	HsDHFR	LmDHFR	PvRdhfr	Lm active	PvR active	Evidence of cross inhibition
TS7_80_4	20110	SM_JHU-520	8	8	0	Yes		Possibly
TS7_80_6	20110	SM_JHU-520	22	20	0	Yes		Possibly
TS7_80_3	20134	SM_JHU-657	20	12	0	Yes		Possibly
TS7_80_4	20248	SM_JHU-1305	8	8	12	Yes	Yes	Possibly
TS7_80_6	20248	SM_JHU-1305	0	8	10	Yes	Yes	No
TS7_80_3	20449	SM_JHU-2317	40	40	0	Yes		Possibly
TS7_80_3	20472	SM_JHU-2438	22	16	0	Yes		Possibly
TS7_80_3	21619	SM_JHU-10190	40	40	16	Yes	Yes	Possibly
TS7_80_3	21767	SM_JHU-12096	0	0	16		Yes	No
TS7_80_6	21767	SM_JHU-12096	32	36	32	Yes	Yes	Possibly

Table S15. NMT assay 1, JHCCL compounds

	Eve ID	JHCCL ID	HsNMT	TbNMT	PvNMT	Tb active	Pv active	Evidence of cross inhibition
NMT_78_6	20110	SM_JHU-520	14	8	8	Yes	Yes	Possibly
NMT_78_4	20248	SM_JHU-1305	16	24	24	Yes	Yes	Possibly
NMT_78_6	20248	SM_JHU-1305	4	18	12	Yes	Yes	No
NMT_78_6	20414	SM_JHU-2095	16	19	19	Yes	Yes	Possibly
NMT_78_2	20470	SM_JHU-2430	1	24	20	Yes	Yes	No
NMT_78_2	20484	SM_JHU-2524	4	28	26	Yes	Yes	No
NMT_78_2	20548	SM_JHU-2987	8	16	16	Yes	Yes	Possibly
NMT_78_2	20626	SM_JHU-3353	0	32	2	Yes		No
NMT_78_2	20633	SM_JHU-3415	0	10	0	Yes		No
NMT_78_2	21006	SM_JHU-6047	0	32	0	Yes		No
NMT_78_6	21006	SM_JHU-6047	0	40	30	Yes	Yes	No
NMT_78_2	21400	SM_JHU-8555	1	20	14	Yes	Yes	No
NMT_78_6	21919		0	22	0	Yes		No

Table S16. NMT assay 2, JHCCL compounds

	Eve ID	JHCCL ID	HsNMT	SmNMT	TcNMT	Sm active	Tc active	Evidence of cross inhibition
NMT_79_6	20110	SM_JHU-520	16	11	11	Yes	Yes	Possibly
NMT_79_6	20168	SM_JHU-904	3	0	17		Yes	No
NMT_79_2	20245	SM_JHU-1293	0	8	10	Yes	Yes	No
NMT_79_6	20248	SM_JHU-1305	8	14	12	Yes	Yes	Possibly
NMT_79_6	20414	SM_JHU-2095	20	21	20	Yes	Yes	Possibly
NMT_79_2	20449	SM_JHU-2317	40	40	40	Yes	Yes	Possibly
NMT_79_2	20472	SM_JHU-2438	26	24	20	Yes	Yes	Possibly
NMT_79_2	20525	SM_JHU-2831	0	16	0	Yes		No
NMT_79_2	20626	SM_JHU-3353	4	40	40	Yes	Yes	No
NMT_79_6	21006	SM_JHU-6047	0	40	0	Yes		No
NMT_79_2	21006	SM_JHU-6047	0	40	40	Yes	Yes	No
NMT_79_2	21281	SM_JHU-7761	0	0	16		Yes	No
NMT_79_2	21390	SM_JHU-8513	10	8	10	Yes	Yes	Possibly
NMT_79_2	21463	SM_JHU-9003	0	2	32		Yes	No
NMT_79_6	21919		12	16	10	Yes	Yes	Possibly

Table S17. PGK assay 1, JHCCL compounds

	Eve ID	JHCCL ID	HsPGK	SmPGK	TcPGK	Sm active	Tc active	Evidence of cross inhibition
PGK1_72_2	20070	SM_JHU-327	30	0	26		Yes	Possibly
PGK1_72_2	20110	SM_JHU-520	31	32	30	Yes	Yes	Possibly
PGK1_72_2	20460	SM_JHU-2381	30	29	29	Yes	Yes	Possibly
PGK1_72_2	20635	SM_JHU-3437	78	80	25	Yes	Yes	Possibly
PGK1_72_2	20655	SM_JHU-3636	6	0	20		Yes	No
PGK1_72_2	20750	SM_JHU-4214	64	74	72	Yes	Yes	Possibly
PGK1_72_2	21088	SM_JHU-6452	80	80	18	Yes	Yes	Possibly
PGK1_72_2	21178	SM_JHU-7015	28	64	25	Yes	Yes	Possibly
PGK1_72_2	21224	SM_JHU-7333	70	80	32	Yes	Yes	Possibly

Table S18. PGK assay 2, JHCCL compounds

	Eve ID	JHCCL ID	HsPGK	TbPGK	PvPGK	Tb active	Pv active	Evidence of cross inhibition
PGK2_74_2	20110	SM_JHU-520	6	8	7	Yes		No
PGK2_74_2	20134	SM_JHU-657	17	16	16	Yes	Yes	Possibly
PGK2_74_2	20291	SM_JHU-1539	40	40	40	Yes	Yes	Possibly
PGK2_74_2	20427	SM_JHU-2151	26	24	24	Yes	Yes	Possibly
PGK2_74_2	20750	SM_JHU-4214	37	33	32	Yes	Yes	Possibly
PGK2_74_2	21088	SM_JHU-6452	40	38	22	Yes	Yes	Possibly
PGK2_74_2	21224	SM_JHU-7333	40	40	38	Yes	Yes	Possibly

7. *In vitro* Enzyme Assays

Materials and Methods

TNP-470 was purchased from Sigma-Aldrich.

Purification of DHFRs

Fresh overnight culture from a single colony of *E. coli* BL21(DE3)pLysS cells harboring wild-type PvDHFR (20) and human DHFR were used to inoculate 1-4 liters of LB medium supplemented with 100 μg/ml ampicillin. The culture was grown at 37˚C until OD_{600} reached 0.5-0.6, at which time the expressions of PvDHFR and human DHFR were initiated by addition of IPTG at a final concentration of 0.4 mM. The culture was allowed to grow with vigorous shaking at 20˚C for additional 18-20 h prior to harvesting by centrifugation at 10,000 g for 10 min at 4˚C. The cell pellet was washed once with ~200-250 mL of cold phosphate-buffered saline, followed by re-suspending in ~20-50 mL of buffer A (20 mM potassium phosphate buffer, pH 7.0, 0.1 mM EDTA, 10 mM DTT). The cells were then disrupted by two cycles of French press at 18,000 psi. The clear extract obtained after centrifugation at 30,000 g for 1 h at 4˚C was applied onto an MTX-Sepharose CL-6B affinity column (1.5 x 5.0 cm), and the enzymes were affinity purified according to the procedure described previously (21).

Enzyme assay and Inhibition Studies

The activity of DHFR was determined spectrophotometrically by monitoring the rate of decrease in absorbance at 340 nm (22, 23). The standard DHFR assay (1 ml) performed in 1-cm path-length cuvette contained 1x DHFR buffer (50 mM TES, pH 7.0, 75 mM β-mercaptoethanol, 1 mg/mL bovine serum albumin), 100 μM H_2folate, 100 μM NADPH, and ~0.01 units of affinity-purified enzyme. The reaction was initiated with H_2folate. One unit of DHFR activity is defined as the amount of enzyme that produces 1 μmole of product per minute at 25 °C. Inhibition studies were carried out by including increasing amounts of the tested compounds in the assay reaction. The reaction was initiated with H_2folate, and the decrease in absorbance at 340 nm was monitored as mentioned above.

Results

**Compound**	**IC_{50}**	
	***P. vivax* DHFR (wild-type)**	**Human DHFR**
TNP-470	0.16 μM	No inhibition (≥ 165 μM)

Table S19. Inhibition by TNP-470 of wild type *P. vivax* DHFR compared to human DHFR.

8. References

1. Elizabeth Bilsland, Pinar Pir, Alex Gutteridge, Alexander Johns, Ross D King, and Stephen G Oliver. Functional expression of parasite drug targets and their human orthologs in yeast. *PLoS neglected tropical diseases*, 5(10):e1320, 2011.
2. Sabine Keppler-Ross, Christine Noffz, and Neta Dean. A new purple fluorescent color marker for genetic studies in saccharomyces cerevisiae and candida albicans. *Genetics*, 179(1):705-710, 2008.
3. Mark A Sheff and Kurt S Thorn. Optimized cassettes for fluorescent protein tagging in saccharomyces cerevisiae. *Yeast*, 21(8):661-670, 2004.
4. Andrew Sparkes, Wayne Aubrey, Emma Byrne, Amanda Clare, Muhammed N Khan, Maria Liakata, Magdalena Markham, Jem Rowland, Larisa N Soldatova, Kenneth E Whelan, et al. Review towards robot scientists for autonomous scientific discovery. *Autom Exp,* 2, 2010.
5. R. D. King, J. Rowland, S. G. Oliver, M. Young, W. Aubrey, E. Byrne, M. Liakata, M. Markham, P. Pir, L. N. Soldatova, A. Sparkes, K. E. Whelan, and A. Clare. The automation of science. *Science*, 324(5923):85-89, 2009.
6. R. D. King, K. E. Whelan, F. M. Jones, P. G. K. Reiser, C. H. Bryant, S. H. Muggleton, D. B. Kell, and S. G. Oliver. Functional genomic hypothesis generation and experimentation by a robot scientist. *Nature*, 427(6971):247-252, 2004.
7. Mark Hall, Eibe Frank, Geoffrey Holmes, Bernhard Pfahringer, Peter Reutemann, and Ian H Witten. The weka data mining software: an update. *ACM SIGKDD Explorations Newsletter*, 11(1):10-18, 2009.
8. I.H. Witten and E. Frank. Data Mining: *Practical machine learning tools and techniques*. Morgan Kaufmann Pub, 2005.
9. C.M. Bishop et al. *Pattern recognition and machine learning*. Springer New York:, 2006.
10. Rajarshi Guha. Chemical informatics functionality in r. *Journal of Statistical Software*, 18(5):1-16, 2007.
11. K. De Grave, J. Ramon, and L. De Raedt. Active learning for primary drug screening. *status: published*, 2008.
12. Pieter Vandezande, Lieven EM Gevers, Johan S Paul, Ivo FJ Vankelecom, and Pierre A Jacobs. High throughput screening for rapid development of membranes and membrane processes. *Journal of membrane science*, 250(1):305-310, 2005.
13. Donald R Jones, Matthias Schonlau, and William J Welch. Efficient global optimization of expensive black-box functions. *Journal of Global optimization*, 13(4):455-492, 1998.
14. Dennis D Cox and Susan John. Sdo: A statistical method for global optimization. *Multidisciplinary design optimization: state of the art*, pages 315-329, 1997.
15. Kurt De Grave, Jan Ramon, and Luc De Raedt. Active learning for high throughput screening. In *Discovery Science*, pages 185-196. Springer, 2008.
16. Noel M OBoyle, Michael Banck, Craig A James, Chris Morley, Tim Vandermeersch, and Geoffrey R Hutchison. Open babel: An open chemical toolbox. *Journal of cheminformatics*, 3(1):1-14, 2011.
17. Craig A James, D Weininger, and J Delany. Daylight theory manual. *Daylight chemical information systems*, 3951, 1995.

18. D.Weininger. SMILES 1. Introduction and encoding rules. *J. Chem. Inf. Comput. Sci*, 28:31, 1988.
19. D. Weininger, A. Weininger, and J.L. Weininger. SMILES. 2. Algorithm for generation of unique SMILES notation. *Journal of Chemical Information and Computer Sciences*, 29(2):97-101, 1989.
20. U. Leartsakulpanich, M. Imwong, S. Pukrittayakamee, N. J. White, G. Snounou, W. Sirawaraporn, Y. Yuthavong. Molecular characterization of dihydrofolate reductase in relation to antifolate resistance in *Plasmodium vivax*. *Molecular and Biochemical Parasitology*, 2002, **119**: 63-73.
21. W. Sirawaraporn, P. Prapunwattana, R. Sirawaraporn, Y. Yuthavong, D. V. Santi. The dihydrofolate reductase domain of *Plasmodium falciparum* thymidylate synthase-dihydrofolate reductase. Gene synthesis, expression, and anti-folate-resistant mutants. *J Biol Chem*. 1993, **268**: 21637-21644.
22. C. B. Brachmann, A. Davies, G. J. Cost, E. Caputo, J. Li, P. Hieter, J. Boeke. Designer deletion strains derived from *Saccharomyces cerevisiae* S288C: a useful set of strins and plasmids for PCR-mediated gene disruption and other applications. *Yeast* 14, 115 (Jan 30, 1998).
23. E. Gari, L. Piedrafita, M. Aldea, E. Herrero. A set of vectors with a tetracycline-regulatable promoter system for modulated gene expression in *Saccharomyces cerevisiae*. *Yeast* 13, 837 (Jul, 1997).
